# Supplementary material for: Melanopsin-Driven Light Adaptation in Mouse Vision
Source: Curr Biol. 2014 Nov 3;24(21):2481–90. doi: 10.1016/j.cub.2014.09.015 (PMC4228053; doi:10.1016/j.cub.2014.09.015)
Supplement: Document S1. Supplemental Experimental Procedures and Figures S1–S4 [file mmc1.pdf]

Current Biology, Volume *24*

Supplemental Information

Melanopsin-Driven Light Adaptation in Mouse Vision

Annette E. Allen, Riccardo Storchi, Franck P. Martial, Rasmus S. Petersen, Marcelo A. Montemurro, Timothy M. Brown, and Robert J. Lucas

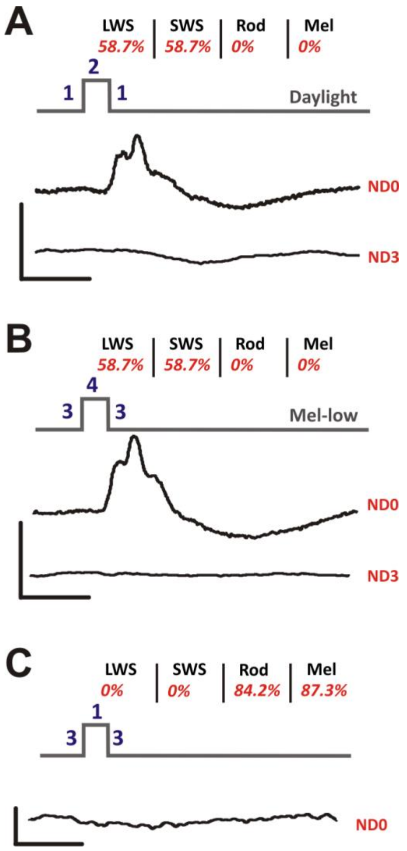

Figure S1. Further validation of spectra used to generate 'daylight' and 'mel-low' flash stimuli. a&b.) Relating to Figure 1. The flash stimuli presented under ódaylightôand ómel-lowô conditions were designed to be visible to cone but not rod (or melanopsin) photoreceptors. As a first confirmation that this was the case, we recorded ERGs at a background light intensity (ND3) 1000 fold lower than that used to generate the data presented in Figs 2 and 3 (ND0). Rods should be more, and cones, less active at this lower irradiance and we found that neither ódaylightô nor ómel-lowô stimuli evoked a measurable ERG under these conditions. Representative responses from a single animal are plotted in a&b. **c.)** The spectra used to generate background illumination under ódaylightôand ómel-lowôconditions (spectra 1 and 3 in Fig. 1 respectively) were designed to be isoluminant for cones. To determine whether this was the case, we attempted to record ERGs to transitions between the two spectra (a 50ms flash of spectrum 1 presented against a background of spectrum 3, at 1Hz; 200 repeats). This stimulus failed to elicit a measurable ERG at the irradiances used for these experiments in all 7 mice tested, consistent with the prediction that they are cone isoluminant. C shows a representative response from a single animal. Flash stimuli are shown in cartoon form above the ERG traces with the spectrum used to produce each element of the stimulus shown in blue (see Fig. 1 for spectral power distributions). Predicted Michelson contrasts for transitions between the stimulus pairs for each photoreceptor are given in red text above.

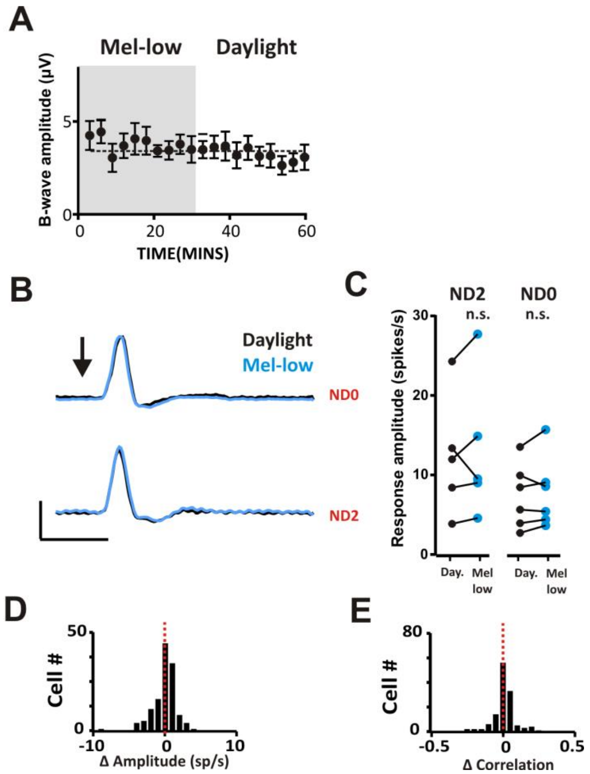

Figure S2. Retinal and dLGN responses to ‘daylight’ and ‘mel-low’ flash stimuli are indistinguishable in melanopsin knockout mice. Relating to Figures 2&3. **a.)** ERG response amplitudes recorded in 8 *Opn4*$^{-/-}$*; Opn1mw*^{R} mice exposed to repeated flashes across the transition from ómel-lowô to ódaylightô conditions; graph shows mean±SEM. These data were collected in parallel with those for *Opn1mw*^{R} data shown in Fig. 2c and compared by two-way ANOVA which revealed significant effects of genotype and genotype/time interactions ($p<0.05$); post-hoc Bonferonni multiple comparisons tests against data for that genotype at time zero revealed significant differences for many of the ódaylightô recordings in *Opn1mw*^{R} mice (Fig. 2c) but not for any time point in melanopsin knockouts. **b.)** The change in multi-unit firing across recording sites in the dLGN of a representative *Opn4*$^{-/-}$*; Opn1mw*^{R} mouse in response to ódaylightô (black line) and ómel-lowô (blue) stimuli at ND0 and ND2. Scale bars: x=250ms, y =10spikes/s; stimuli as for Fig. 4; arrow depicts time of flash. **c.)** The mean change in multiunit firing rate in the 200ms following flash across recording sites in the dLGN of *Opn4*$^{-/-}$*; Opn1mw*^{R} mice was not significantly different between ódaylight (black symbols) and ómel-lowô (blue) at either ND0 or ND2 ($p>0.05$ paired t-tests. **d.)** The distribution of differences in this measure of response amplitude between conditions (ómel-lowô response ï ódaylightô response) for single units from *Opn4*$^{-/-}$*; Opn1mw*^{R} mice fails to recapitulate the shift in this parameter observed for *Opn1mw*^{R} mice (Fig. 3f). **e.)** The distribution of difference in autocorrelation (Pearsonõs correlation coefficient) in the response of single units between conditions (ómel-lowô ï ódaylightô) also shows that these melanopsin knockouts lack the change in this parameter reported for *Opn4*$^{-/-}$*; Opn1mw*^{R} mice (Fig. 3g).

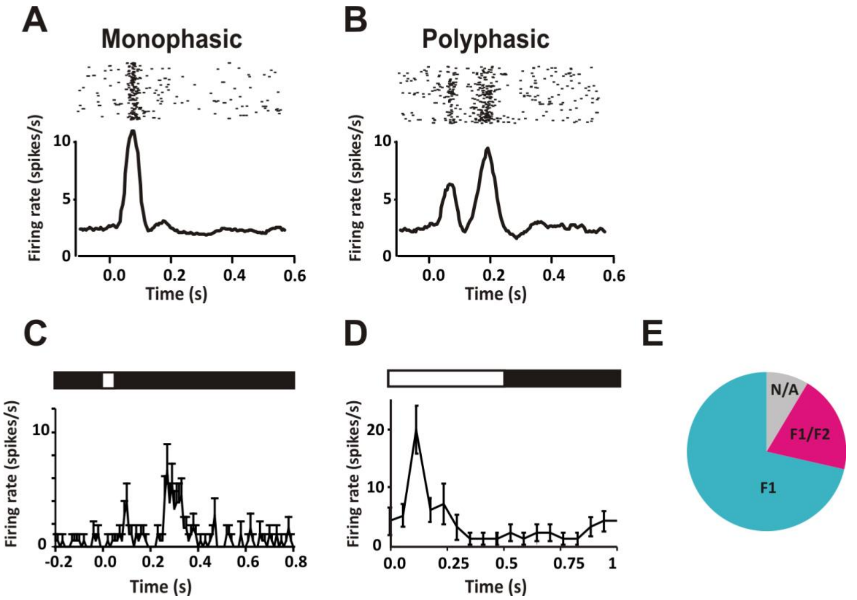

Figure S3. Exploring temporal changes in flash response profiles. Relating to Figures 3&4. There was no change in response latency between conditions, with the initial peak in firing lagging the flash by 89.1 ± 4.4msec in ómel-lowô and 81.8 ± 5.28msec in ódaylightô (mean±SEM; paired two-way T-test p=0.14). However, under either condition responses could be regarded as falling into two broad categories, in the first (termed ómonophasicö) there was a single peak in firing around 50ms after the flash (**a**), in the second (ópolyphasicö) this first peak was followed by one or more additional peaks over the 200ms post flash period (**b**). Above are peri-event rasters for 200 stimulus repeats, with associated peri-event time histograms below (flash onset at time 0). The two categories were sufficiently distinct to allow them to be defined by subjective assessments, but we confirmed the distinction using principle component analysis. Under any single background lighting condition the response of individual units did not change, but it was common to see units switch categories between ódaylightô and ómel-lowô conditions (Fig. 3d). In *Opn1mw*^{R} mice there was a net shift towards polyphasic responses when switching to the ódaylightô spectrum with 19% of all light responsive cells changing from monophasic to polyphasic. This effect was absent in *Opn4*$^{-/-}$*; Opn1mw*^{R} mouse confirming that it was driven by melanopsin. We asked whether the polyphasic response type was indicative of óON-OFFô response types by examining their responses to inverting gratings. **c&d** show representative responses of a polyphasic cell to the ódaylightô flash (**c**) and to inverting gratings (**d**) under ódaylightô conditions at 0.018cpd (stimulus presentation and data acquisition as described for the data in Figs 3 and 4). Note that when presented with the inverted grating this unit responds only to the increase in luminance over its receptive field centre indicating that it is an óONô cell. If there were a preponderance of ON-OFF responses for polyphasic units, one would expect to see frequency doubled responses to the inverted gratings (i.e. units responding to the appearance of white and black bars). To determine whether this was the case, the amplitude of dominant first and second harmonics (F1 and F2, corresponding to the 2Hz reversal frequency of the grating and 4Hz frequency doubling) were extracted from Fourier transforms of single unit responses to inverting gratings in the preferred orientation and phase. F1 and F2 were designated as significant if they lay outside 2 standard deviations of the Fourier transform of a shuffled version of the response. **e.)** A pie chart of the number of polyphasic units showing F1, both F1 and F2, no significant response (N/A) to the inverted

gratings indicates that most units designated as polyphasic lack the F2 component expected of óON-OFFô cells. Polyphasic cells did not differ in either preferred temporal or spatial frequency, nor were they more or less likely to be direction sensitive. Thus, while there clearly were changes in temporal profile of flash responses between conditions, and while these provide further evidence of melanopsin-dependent adjustments in the visual code, we cannot at present determine what significance this event has for encoding more natural visual stimuli.

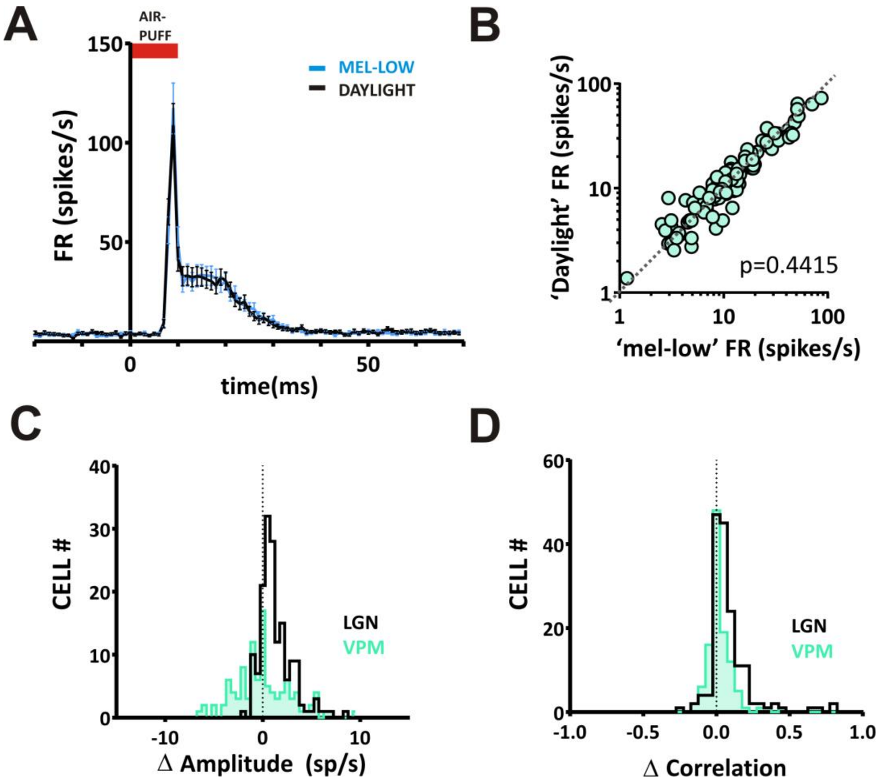

Figure S4. Changes in response in 'daylight' and 'mel-low' condition are specific to visual thalamus, and do not reflect a non-specific change in neuronal activity. Relating to Figures 4-6. **a&b.)** The amplitude of responses evoked by whisker movement (1Hz 10ms rostro-caudal air-puff stimulus), recorded in the ventral posteromedial region of the somatosensory thalamus (VPM), were also assessed in the ómel-lowô and ódaylightô conditions. No change in the response amplitude was detectable. **a,** the mean ±SEM firing rate of 112 units, recorded in 7 *Opn1mw*^{R} mice in each condition. **b,** a scatter plot of the response amplitude (change in FR in first 20ms of response) in each condition; paired T-test finds no change in amplitude between conditions (p=0.4415). **c&d,** A direct comparison of the distribution of the change in amplitude (**c**) and correlation (**d**), between ómel-lowô and ódaylightô conditions, in cells recorded in the dLGN (black; re-plotted from Fig.4) and VPM (green). Note that cells recorded in VPM are unaffected by the change in background, unlike those from dLGN.

SUPPLEMENTAL EXPERIMENTAL PROCEDURES

Electroretinography

ERGs were recorded from thirteen *Opn1mw*^{R} and eight *Opn4*$^{-/-}$*; Opn1mw*^{R} male mice (aged 3-6 months). Mice were from a mixed C57/BL6; 127sv strain. *Opn1mw*^{R} refers to the transgenic allele originally generated by Smallwood *et al*. (2003), and termed ñRò by them [S1]. *Opn4*$^{-/-}$ mice contain an insertion of tau-lacZ into the melanopsin gene locus, rendering mice ómelanopsin-knockoutô [S2]. All animal care was in accordance with the Animals, Scientific Procedures, Act of 1986 (UK). Animals were kept in a 12-hour dark/light cycle at a temperature of 22°C with food and water available *ad libitum*.

Anaesthesia was induced with an intra-peritoneal injection of urethane (1.6g/kg; 30%w/v; Sigma-Aldrich). A topical mydriatic (tropicamide, 1%; Chauvin Pharmaceuticals) and mineral oil (Sigma-Aldrich) were applied to the recording eye prior to placement of a corneal contact-lens type electrode. Mice were placed into a stereotaxic frame to keep a fixed head-position; a bite bar was also used for head support, and acted as a ground. A needle reference electrode (Ambu, Neuroline) was inserted approximately 5mm from the base of the contralateral eye. Electrodes were connected to a Windows PC via a signal conditioner (Model 1902 Mark III, CED) that differentially amplified (X3000) and filtered (band-pass filter cut off 0.5 to 200Hz) the signal, and a digitizer (Model 1401, CED). Core body temperature was maintained at 37°C throughout recording with a homeothermic heat mat (Harvard Apparatus). B-wave amplitudes were measured relative to baseline values (time of flash onset), since a-waves were not readily measurable

In vivo physiology

Neuronal activity within the dLGN to the flash stimuli was recorded concurrently with ERGs. In addition, a separate set of mice (six *Opn1mw*^{R} and three *Opn4*$^{-/-}$*; Opn1mw*^{R} male mice, aged 3-6 months) was used to record responses to spatially structure stimuli. After placement into the stereotaxic frame, the mouse's skull surface was exposed and a small hole drilled ~2.3mm posterior and ~2.3mm lateral to the bregma. A recording probe (A4x8-5mm-50-200-413; Neuronexus) consisting of 4 shanks spaced 200µm apart, each with 8 recording sites (spaced 50µm, sized 413µm^2), was lowered a depth of ~2.5-3mm into the brain, targeting the dorsal lateral geniculate nucleus (dLGN). In addition, in 7 *Opn1mw*^{R} mice, recordings were made in the ventral posteromedial region of the somatosensory thalamus (VPM; -1.8 - 2.1mm posterior and 1.4mm lateral to bregma; lowered ~3.5mm). Neural signals were acquired using a Recorder64 system (Plexon), and were amplified (x3000), highpass filtered (300Hz), and digitised at 40kHz. Multiunit activity was saved and analysed offline using Offline Sorter (Plexon). After removing artefacts common to all channels, principal component analyses were used to discriminate single units, identified as distinct clusters of spikes within the principal component space, with a clear refractory period in the interspike interval distribution. Spike sorted data were then further analysed using Neuroexplorer (Nex Technologies) and MATLAB R2010a (The Mathworks Inc.), to assess the changes in firing rate of neurons in response to different visual stimuli.

Histology

To establish the location of recording sites, the recording electrode was dipped in fluorescent dye (Cell Tracker CM-DiI; Invitrogen) prior to insertion. In other experiments we have found good correspondence between electrode placements reconstructed using this method and by use of electrolytic lesions [S3]. Following recordings, the mouseôs brain was removed and

post-fixed overnight in 4% paraformaldehyde, prior to cryoprotection for 24 hours in 30% sucrose. 99εm coronal sections were then cut using a sledge microtome, mounted onto glass slides and cover slips were applied using Vectashield (Vector Laboratories, Inc.).

Visual stimuli

Light calibration: Stimuli were measured at the corneal plane using a spectroradiometer (Bentham Instruments Ltd.) between 300-800nm. The effective photon flux for each photopigment was then calculated by weighting spectral irradiance according to pigment spectral efficiency profile as estimated by the pigment spectral efficiency function (derived from a visual pigment template [S4] and ə$_{max}$ values of 365, 480, 498, 508 and 556nm for SWS opsin, melanopsin, rod opsin, MWS opsin and the introduced LWS opsin respectively) mutliplied by an *in vivo* measurement of spectral lens transmission [S5]. The approach is equivalent to that described in Lucas et al (2014) [S6], using spectral efficiency functions available at:
http://lucasgroup.lab.ls.manchester.ac.uk/research/measuringmelanopicilluminance.

Full field visual stimuli: Full field visual stimuli were generated using a custom-made light source (Cairn Research) containing three independently controlled LEDs (ə$_{max}$ at 365nm, 460nm and 600nm). Light from LEDs was combined by a series of dichroic mirrors, passed through a filter-wheel containing neutral-density filters and focused onto opal diffusing glass (5mm diameter; Edmund Optics Inc.) positioned <1mm from the eye. LED intensities and the filter wheel position were controlled with a PC running LabView 8.6 (National Instruments, Ltd.). LEDs were combined to generate two background and stimulus combinations that are summarised in Fig. 1d. The effective photons for ómel-lowô and ódaylightô backgrounds were calculated to be cone isoluminant (14.6 log$_{10}$ LWS-opsin effective photons/cm^2/s (3.0 log$_{10}$ erythropic-lux); 13.9 log$_{10}$ SWS-opsin effective photons/cm^2/s (2.8 log$_{10}$ cyanopic-lux)), but divergent for rods and melanopsin (14.0 and 15.1 log$_{10}$ rod-opsin effective photons/cm^2/s (2.6 and 3.6 log$_{10}$ rhodopic-lux); 14.0 and 15.2 log$_{10}$ melanopsin effective photons/cm^2/s (2.5 and 3.7 log$_{10}$ melanopic-lux)). óMel-lowô and ódaylightô stimuli were generated that were isoluminant to ómel-lowô and ódaylightô backgrounds for rods and melanopsin, but which presented contrast for cone opsins (stimulus = 15.2 log$_{10}$ LWS-opsin effective photons/cm^2/s (3.5 log$_{10}$ erythropic-lux); 14.5 log$_{10}$ SWS-opsin effective photons/cm^2/s (3.3 log$_{10}$ cyanopic-lux)),

Patterned stimuli: Structured images were presented using a custom-made light source containing four independently controlled LEDs (ə$_{max}$ at 405nm, 455nm, 525nm and 630nm; Phlatlight PT-120 Series (Luminus Devices)). LED intensities were controlled with a PC running LabView v.12 (National Instruments, Ltd.). Light from the LEDs was combined by a series of dichroic mirrors (Thorlabs), and directed into a digital mirror device projector (DLP® LightCommanderÊ; Logic PD Inc.) in place of the original intrinsic light source. Artificial images were generated using Python running PsychoPy Version 1.70.00 [S7].

To expand the area of the retina exposed to the ódaylightô and ómel-lowô spectra additional LED lighting surrounded the projection screen. These LEDs (peak emission at 400nm (Component-Shop), 460nm, 517nm and 630nm (LEDLightsZone)) were arranged in a high density array, and were placed behind Opal Polypropylene (2mm thickness; The Plastic People) to create a diffuse surround. LED intensities were controlled with a Micro-controller

(Arduino UNO, Creative Commons), and matched equivalent photon fluxes of the projection screen.

Although the spectral composition of LEDs in the projector and the surround were thus different from those used for the full field flash, we were able to recreate the effective photon flux for each photopigment of the original ódaylightô and ómel-lowô stimuli based upon spectral irradiance measures at the corneal plane. The radiance of the projected image was also measured, and resulted in intensities of 14.5 and 13.3 log_{10} melanopsin effective photons/cm^2/sr/s (3.0 and 1.8 log_{10} melanopic lm/sr), in ódaylightô and ómel-lowô backgrounds, respectively.

Before presenting structured images with this apparatus, we first confirmed its suitability for exploring melanopsin regulation of vision by duplicating our findings from the full-field flash stimuli. Thus, we projected the cone-isolating flash stimuli under ómel-lowô and ódaylightô conditions across the surface of the screen. The proportion of dLGN units responding to this stimulus was smaller than for full field presentations, as expected given the reduced coverage of the visual scene. In agreement with our earlier data, we found that in *$Opn1mw^R$* mice the amplitude of the mean response was increased in the ómel-lowô condition (paired t-test of light-evoked change in firing rate, p=0.011). Importantly, *$Opn4^{-/-}$;$Opn1mw^R$* animals showed no consistent difference in flash responses between ódaylightô and ómel-lowô conditions (p>0.05 paired t-test).

Subsequently, responses to a range of stimuli were recorded first in the ómel-lowô, then ódaylightô, then the ómel-lowô spectra again, to ensure no temporal drift in response properties. To assay changes in spatial frequency tuning, inverting gratings (Michelson contrast between dark and light bars = 96%) were presented in 4 orientations at two phases (shifted 90°), at 5 different spatial frequencies (0.035-0.56cpd) at 1Hz. To assay changes in temporal frequency tuning, drifting gratings in 8 orientations, at four speeds (0.2-2Hz), were presented at one spatial frequency (0.035cpd). To map receptive fields, bars of positive and negative contrast (Michelson contrast = 72.7%) were presented in the vertical, then horizontal orientation (occupying ~4.5 and 3.6 degrees of the visual field, respectively). These were presented in a pseudo-randomised sequence at 20Hz. Spatial receptive fields were then derived from the responses to this sequence (see below). Responses to a 30s natural movie were also recorded. The natural movie was a recording of mice moving around a behavioural arena, and included movement and looming of different sized objects (subtending visual angles ranging from 0.5 to 36°) at a range of orientations, speeds and contrasts (maximum Michelson contrast = 96 %). The movie lacked differences in colour, and changes in irradiance across time were minimal (standard deviation of irradiance = 5.94%). Responses were undetectable for presentations of de-focussed versions, indicating that most activity was elicited by changes in spatial patterns and object motion.

Environmental light measurements: To gain insight into the range of environmental light levels over which it is appropriate to measure visual responses, we measured the ambient light levels in Manchester, UK (53°21' N, 2°16' W, elevation of 78m), 2 weeks after Summer solstice, for solar angles ranging from ~30 to -9° (equivalent to 1800-2300hrs GMT+1). All solar angles were measured using a spectroradiometer (Bentham Instruments), measuring the relative power in mW/cm^2 at wavelengths between 300-800nm.

Statistical analyses

Receptive field mapping: The spatio-temporal receptive field was derived for each unit by generating the spike triggered average (STA) of responses to bars presented in vertical and horizontal orientations. Black and white bars covering 1/16th of a grey screen were presented at random locations every 50ms (for a duration of 50ms). Bars were preferred to squares on the base of our pilot experiments because they evoked stronger and more repeatable responses, enabling us to obtain more reliable spatiotemporal receptive field estimates and minimize the time of stimulus presentation. The separable spatial and temporal components where then extracted from the raw STA matrix by applying the principal components analysis to rows and columns. The eigenvectors associated with the largest eigenvalues were taken as spatial and temporal kernels. Although the non-separable components of the receptive fields were lost in this procedure, most of energy of the receptive field was preserved with the additional advantage of removing a substantial amount of noise from the raw STA estimates. The spatial receptive field sizes were then separately estimated for the horizontal and vertical dimensions by fitting a Gaussian to the spatial kernels derived from the previous analyses. The receptive field size for individual cells was described as the mean width at half maximum of Gaussians fitted to each dimension. To compare whether spatial receptive fields differed between ódaylightô and ómel-lowô conditions, in each dimension, Gaussians were compared with an F-test, to test whether receptive fields were best fit with a single, or two individual Gaussians.

Classification of direction sensitivity: The direction selectivity index was calculated as described previously [S8]. This was computed as the ratio of $(R_{pref}-R_{null})/(R_{pref}+R_{null})$, where R_{pref} was the response at which the maximum evoked response occurred, and R_{null} was response to movement in the opposite direction to this. Cells exceeding a direction selectivity index of 0.5 were classed as ódirection selectiveô

Natural movie correlation analyses: First, to find cells that showed any kind of reproducible response to the natural movie, we performed a consistency test to the firing rate responses of single units across multiple presentations of the natural movie. Briefly, we computed the average Pearsonõs correlation among pairs of single trial responses. Then we estimated its standard deviation by using a standard bootstrap technique [S9]. We classified a neuronal response as consistent if its average trial-to-trial Pearsonõs correlation was larger than 3 times the standard deviation. Any cell that showed a consistent response, in the ómel-lowô and/or ódaylightô condition, was then included in further analyses. To assess the variation in responses between cells during presentation of the natural movie, we compared the pairwise PSTH Pearsonõs linear correlation (hereafter ósignal correlationô) in the ódaylightô and ómel-lowô conditions. For these cells, we also compared the average trial-by-trial reliability (óautocorrelationô) of responses by computing the Pearsonõs correlation among pairs of single trial responses (time bin = 0.1s) in the ódaylightô and ómel-lowô conditions.

SUPPLEMENTAL REFERENCES

S1. Smallwood, P.M., Olveczky, B.P., Williams, G.L., Jacobs, G.H., Reese, B.E., Meister, M., and Nathans, J. (2003). Genetically engineered mice with an additional class of cone photoreceptors: implications for the evolution of color vision. PNAS *100*, 11706-11711.

S2. Lucas, R.J., Hattar, S., Takao, M., Berson, D.M., Foster, R.G., and Yau, K.W. (2003). Diminished pupillary light reflex at high irradiances in melanopsin-knockout mice. Science *299*, 245-247.

S3. Allen, A.E., Brown, T.M., and Lucas, R.J. (2011). A distinct contribution of short-wavelength-sensitive cones to light-evoked activity in the mouse pretectal olivary nucleus. J Neurosci *31*, 16833-16843.

S4. Govardovskii, V.I., Fyhrquist, N., Reuter, T., Kuzmin, D.G., and Donner, K. (2000). In search of the visual pigment template. Visual Neuroscience *17*, 509-528.

S5. Jacobs, G.H., and Williams, G.A. (2007). Contributions of the mouse UV photopigment to the ERG and to vision. Documenta Ophthalmologica *115*, 137-144.

S6. Lucas, R.J., Peirson, S.N., Berson, D.M., Brown, T.M., Cooper, H.M., Czeisler, C.A., Figueiro, M.G., Gamlin, P.D., Lockley, S.W., O'Hagan, J.B., et al. (2014). Measuring and using light in the melanopsin age. Trends Neurosci *37*, 1-9.

S7. Peirce, J.W. (2007). PsychoPy--Psychophysics software in Python. J Neurosci Methods *162*, 8-13.

S8. Zhao, X., Chen, H., Liu, X., and Cang, J. (2013). Orientation-selective responses in the mouse lateral geniculate nucleus. J Neurosci *33*, 12751-12763.

S9. Efron, B., and Tibshirani, R.J. (1993). An Introduction of the Boostrap, (New York: Chapman and Hall).
