# Supplementary material for: Melanopsin-Driven Light Adaptation in Mouse Vision
Source: Curr Biol. 2014 Nov 3;24(21):2481–90. doi: 10.1016/j.cub.2014.09.015 (PMC4228053; doi:10.1016/j.cub.2014.09.015)
Supplement: Document S2. Article plus Supplemental Information [file mmc2.pdf]

Current Biology 24, 2481–2490, November 3, 2014 ©2014 The Authors http://dx.doi.org/10.1016/j.cub.2014.09.015

Article

Melanopsin-Driven Light Adaptation in Mouse Vision

Annette E. Allen,[1,*] Riccardo Storchi,[1] Franck P. Martial,[1] Rasmus S. Petersen,[1] Marcelo A. Montemurro,[1] Timothy M. Brown,[1] and Robert J. Lucas[1,*]
[1]Faculty of Life Sciences, Manchester University, Oxford Road, Manchester M13 9PT, UK

Summary

Background: In bright light, mammals use a distinct photopigment (melanopsin) to measure irradiance for centrally mediated responses such as circadian entrainment. We aimed to determine whether the information generated by melanopsin is also used by the visual system as a signal for light adaptation. To this end, we compared retinal and thalamic responses to a range of artificial and natural visual stimuli presented using spectral compositions that either approximate the mouse's experience of natural daylight ("daylight") or are selectively depleted of wavelengths to which melanopsin is most sensitive ("mel-low").
Results: We found reproducible and reversible changes in the flash electroretinogram between daylight and mel-low. Simultaneous recording in the dorsal lateral geniculate nucleus (dLGN) revealed that these reflect changes in feature selectivity of visual circuits in both temporal and spatial dimensions. A substantial fraction of units preferred finer spatial patterns in the daylight condition, while the population of direction-sensitive units became tuned to faster motion. The dLGN contained a richer, more reliable encoding of natural scenes in the daylight condition. These effects were absent in mice lacking melanopsin.
Conclusions: The feature selectivity of many neurons in the mouse dLGN is adjusted according to a melanopsin-dependent measure of environmental brightness. These changes originate, at least in part, within the retina. Melanopsin performs a role analogous to a photographer's light meter, providing an independent measure of irradiance that determines optimal setting for visual circuits.

Introduction

The visual system is charged with encoding visual information across the >10^9-fold change in background light intensity from starlight to cloudless midday. The switch between rod- and cone-based vision and adjustments in photoreceptor sensitivity are central to meeting this challenge. However, the behavior of circuits in the retina is also critical, with multiple examples of visual signals being shifted between parallel pathways with different computational characteristics, and of the behavior of individual elements within these pathways changing as a function of irradiance [1, 2]. Such network changes do not merely adjust sensitivity and avoid saturation, but optimize circuit behavior to ensure efficient extraction of visual information (see [3, 4]).

*Correspondence: annette.allen@manchester.ac.uk (A.E.A.), robert.lucas@manchester.ac.uk (R.J.L.)
This is an open access article under the CC BY license (http://creativecommons.org/licenses/by/3.0/).

Effective adaptation relies upon an accurate measure of light intensity. One might expect that adaptation state would be defined by the most accurate available measure of irradiance; under many circumstances, this is provided by a particular class of retinal ganglion cell [5–8]. These intrinsically photosensitive retinal ganglion cells (ipRGCs) have their own melanopsin-dependent phototransduction mechanism [9–11], and employ this, along with extrinsic signals originating with rods and cones, to encode light levels over many decimal orders [6].

The information generated by ipRGCs is exported to the brain where it entrains circadian clocks and sets physiological and behavioral states [12, 13]. The hypothesis that ipRGCs also provide irradiance information to the retina contradicts a standard assumption of retinal function that information flows via ganglion cells to the brain, but not back into the retina. However, there is a growing body of evidence that ipRGCs do not obey this rule. In 2002, it was shown that a diurnal rhythm in an aspect of the human cone electroretinogram (ERG) may be regulated by a photoreceptor with melanopsin-like spectral sensitivity [14]. This was followed by data that revealed ipRGCs make gap-junction connections with neighboring amacrine cells [15, 16] and send axon collaterals to the retinal inner plexiform layer [17]. Meanwhile, there is also physiological evidence that ipRGCs excite dopaminergic amacrine cells, which themselves are influential modulators of retinal circuitry [18].

Establishing whether aspects of network light adaptation really are driven by ipRGCs and what impact (if any) this has on visual function is technically challenging. Historically, the starting point for assigning functions to ipRGCs has been eliminating rod and cone photoreception using genetic or pharmacological approaches [19, 20]. However, such preparations are ill suited to revealing ipRGC influences on conventional vision. Comparing visual responses of wild-type and melanopsin knockout mice could be more informative, but interpreting such data is complicated by evidence that retinal development and retinal circadian rhythms are disrupted in animals lacking melanopsin [21–24].

Here, we therefore set out to explore ipRGC influences on visual responses in animals with an intact retina. Our approach adopts the ideas of metamerism and receptor silent substitution from the field of human psychophysics. In brief, we employed a transgenic mouse (*Opn1mw^R* [25]) in which the spectral sensitivity of cone photoreceptors is substantially shifted compared to that of melanopsin. Using a multispectral light source, we were able to produce background lighting conditions that were equivalent for cones but differed substantially in their effective photon flux for melanopsin. We found substantial differences in retinal and thalamic responses to visual stimuli presented under these two conditions. These differences could be explained, at least in part, by changes in visual feature selectivity of individual units and were associated with alterations in the dorsal lateral geniculate nucleus' (dLGN's) ability to encode natural scenes.

Results

Mouse Cone Metamers

Our strategy for determining whether melanopsin modulates vision was to compare responses to the same visual stimuli

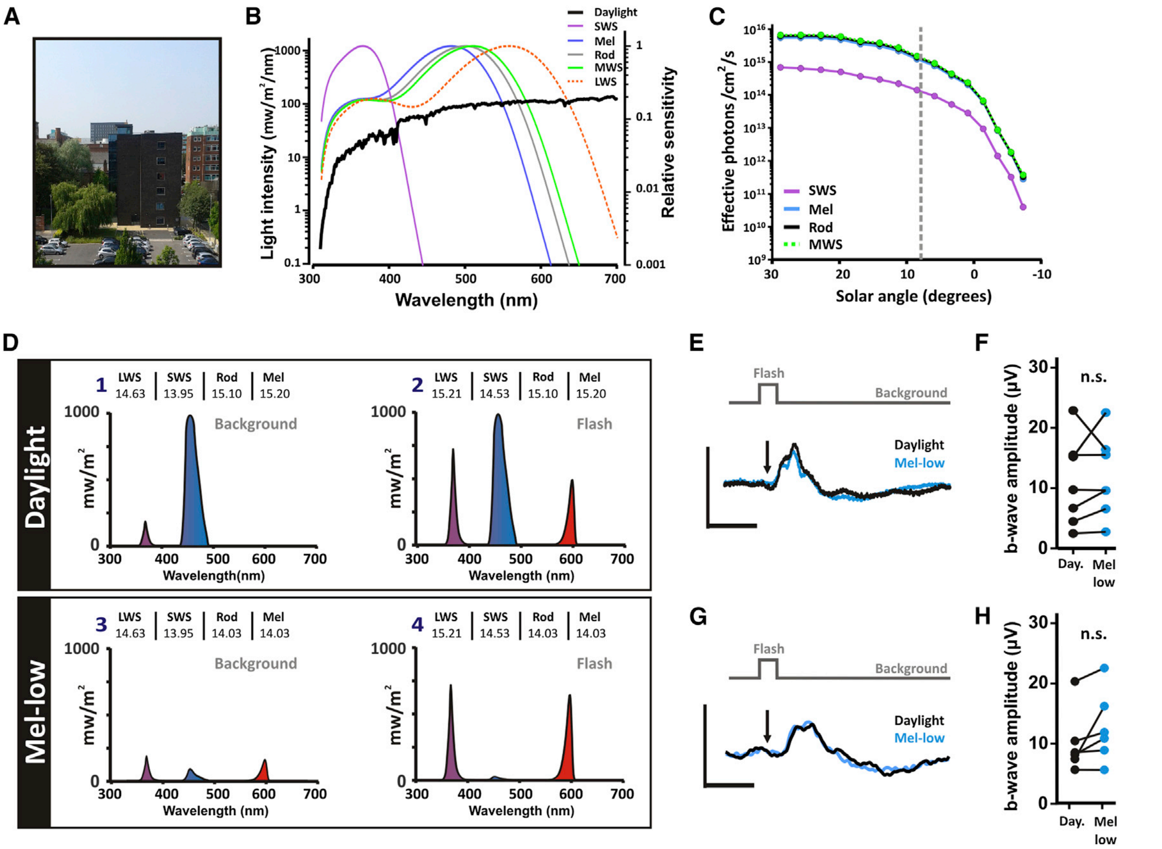

Figure 1. Polyspectral Stimuli Allowing Independent Control of Irradiance as Experienced by Cones versus Melanopsin

(A–H) To ensure that some of our stimuli approximated the mouse's experience of natural light, we recorded spectral irradiance in an urban scene (A) in Manchester over a dusk transition. The resultant spectral power distributions (B, black line shows data for solar angle 11.2°) were multiplied by spectral efficiencies of mouse SWS opsin, melanopsin, rod opsin, and MWS opsin (purple, blue, gray, and green lines, respectively) to calculate effective corneal irradiance in photons/cm^2/s for each photopigment over a range of solar angles (C). To allow independent modulation of cone opsins versus melanopsin, these experiments employed *Opn1mw^R* mice in which mouse MWS opsin is replaced by red-shifted human LWS cone opsin (red dotted line in B). A three primary LED light source (peak emission at 365, 460, and 600 nm) produced four spectrally distinct stimuli shown in (D) with $\log_{10}$ effective photon fluxes for each photopigment in inset. Spectra 1 and 2 approximated the mouse's experience of natural light at solar angle +8°, while spectra 3 and 4 were selectively denuded of those wavelengths to which melanopsin is most sensitive. The individual elements of these daylight and mel-low stimulus pairs were designed to be rod and melanopsin isoluminant but to differ in effective irradiance for SWS opsin and LWS opsin. By contrast, spectra 1 and 3 and spectra 2 and 4 were designed to be cone isoluminant but to differ substantially in effective photon flux for melanopsin and rods. As a result, switching from either spectrum 1 to 2 or spectrum 3 to 4 produced a 58% Michelson contrast step to cones presented against backgrounds differing substantially in effective photon flux for rods and melanopsin. This was validated by measuring ERG responses to 200 repeats of 1 Hz, 50 ms transitions from either spectrum 1 to 2 and back again (daylight) or spectrum 3 to 4 and back (mel-low). Two control ERG measurements were made in response to daylight and mel-low stimuli: (1) in *Opn1mw^R* mice at a moderate intensity (100-fold dimmer than maximum and hence with reduced melanopsin excitation; E and F) and (2) in *Opn4$^{-/-}$;Opn1mw^R* mice at the maximum intensity (G and H). (E) and (G) show representative traces in daylight (black traces) and mel-low (blue traces); arrow indicates time of flash. Scale bars, 100 ms (x); 40 µV (y). Population response amplitudes are plotted in (F) and (H). Data were compared with paired t tests. In each control condition, responses to daylight and mel-low had equivalent amplitudes ($p > 0.05$).

presented against backgrounds appearing equivalent to conventional photoreceptors but differing substantially in effective irradiance for melanopsin. Given the similar spectral sensitivity of mouse medium-wavelength-sensitive (MWS) cone opsin and melanopsin (Figure 1B), to achieve this we employed transgenic mice (*Opn1mw^R*) with a fully intact visual system but in which the red-shifted human long-wavelength-sensitive (LWS) cone opsin is knocked into the MWS cone opsin locus [25] (Figure 1B). A bespoke light source in which the output of three spectrally distinct light-emitting diodes (LEDs) could be independently modulated allowed great scope for generating spectra differing in melanopsin effective photon flux but predicted to be isoluminant ("metameric") for short-wavelength-sensitive (SWS) and LWS cone opsins.

From spectra matching this requirement, we chose two pairs (Figure 1D) that could be used to generate a 50 ms "flash" (stimuli 2 and 4) presented against backgrounds differing in melanopsin photon flux (stimuli 1 and 3). These combinations had the following characteristics. (1) The flash should be visible to cones but not rods or melanopsin. We were interested in modulatory rather than direct contributions of melanopsin to flash responses and thus aimed to make the elements of each background and flash combination melanopsin isoluminant. By working at high irradiances, we hoped to minimize rod influences on our recordings. Nevertheless, as a further precaution, we set background and flash elements to be rod isoluminant. (2) The cone experience of the flash stimulus should be equivalent for the two pairs. Setting both backgrounds and both flashes isoluminant for both SWS and

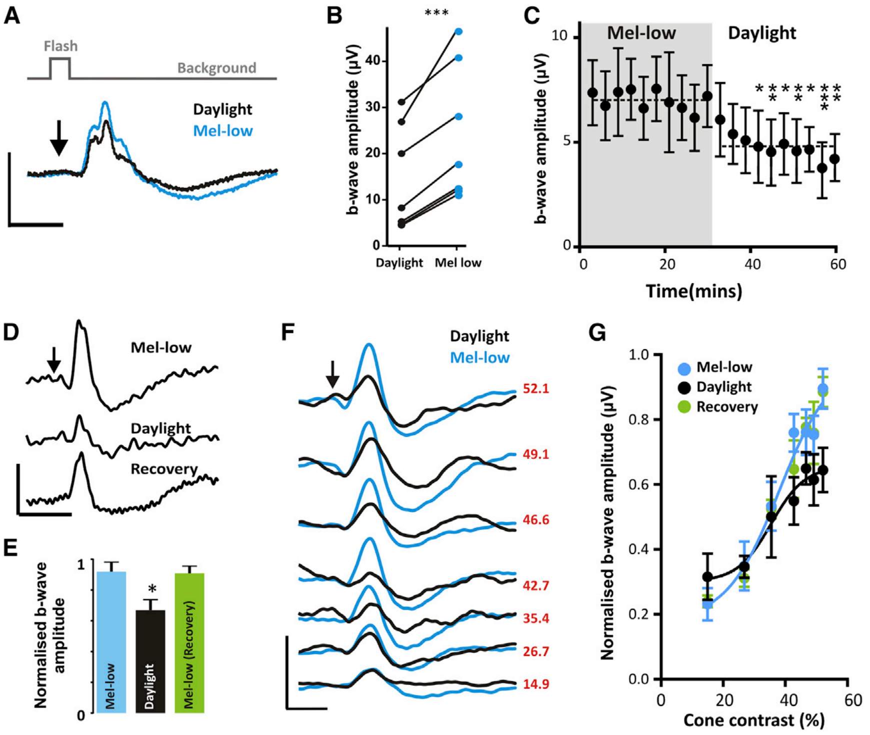

Figure 2. Melanopsin-Dependent Changes in the Cone ERG

(A and B) ERG traces (A) from a representative *Opn1mw*^{R} mouse to 200 repeats of 1 Hz, 50 ms daylight (black traces) or mel-low (blue traces) flashes at maximum intensity (irradiances as in Figure 1D). Arrow indicates time of flash. Scale bars, 100 ms (x); 40 μV (y). The increase in b-wave amplitude apparent in the mel-low condition in this representative trace was observed in all seven *Opn1mw*^{R} mice for which data were recorded (B, black symbols represent b-wave amplitude in daylight condition and blue circles in mel-low condition; paired t test, $p > 0.001$).

(C) The change in ERG b-wave occurred gradually following the switch from mel-low to daylight conditions. Data show mean ± SEM of b-wave amplitude to 180 repeats of the 1 Hz flash stimulus in seven mice. These data were collected in parallel with those for *Opn4*$^{-/-}$;*Opn1mw*^{R} data shown in Figure S2A and compared by two-way ANOVA (significant effects of genotype and genotype-time interactions [$p < 0.05$]; post hoc Bonferroni multiple comparisons tests against *Opn1mw*^{R} at time zero revealed significant differences for daylight recordings after approximately 9 min [*$p < 0.05$, **$p < 0.01$, ***$p < 0.001$]).

(D and E) Transitions from mel-low to daylight and then back again confirmed that the changes in response were reversible; shown for ERG traces from a representative mouse (D) and for normalized b-wave amplitude from six mice (E; mean ± SEM; one-way ANOVA with a post hoc Bonferroni multiple comparisons test: mel-low versus daylight, $p < 0.05$; mel-low versus mel-low [recovery], $p > 0.05$).

(F and G) The contrast response relationship differed between daylight and mel-low conditions. ERG traces (F) from a representative mouse exposed to variants of daylight and mel-low stimuli in which the increase in effective cone excitation of the flash (spectra 2 and 4) was altered (Michelson contrasts provided at right) while maintaining rod and melanopsin isoluminance. Scale bars, 100 ms (x); 20 μV (y). Population contrast response relationships (G) were produced by plotting mean ± SEM b-wave amplitude (normalized to 1 = the maximum recorded for that mouse under any condition; n = 6) against flash cone contrast revealed differences between conditions (F-test comparisons of sigmoidal fits to data; curves for mel-low and daylight conditions are significantly different [$p < 0.05$], but those for mel-low and mel-low [recovery] are not [$p = 0.93$]).

LWS cone opsins across the pairs ensured that the two stimulus conditions were equivalent for each individual cone irrespective of its relative expression of the two pigments [26]. (3) One of the conditions approximates the mouse's experience of natural daylight. We recorded spectral irradiance profiles in horizontal view over a dusk transition in an urban setting (solar angles from −9° to 30° under clear skies but outside of direct sunlight; Figures 1A and 1B) and modeled the mouse's experience of these conditions by calculating the effective photon flux for each of the mouse photopigments (Figure 1C). At all positive solar elevations, the effective photon flux (melanopsin, rod opsin, and MWS opsin) was roughly equivalent and ~10 times greater than that for SWS opsin. Our first stimulus pair (spectra 1 and 3) maintained these activity ratios and recreated the mouse's experience of a solar angle of +8° on our representative day. We therefore refer to this condition as "daylight." In the other stimulus pair (spectra 2 and 4; termed "mel-low"), the effective photon flux for melanopsin was selectively reduced by 10 times.

As these experiments rely upon the daylight and mel-low conditions appearing equivalent to mouse cones (at least within the resolution of our methods of assaying visual response), we first undertook control experiments to confirm that this was true (see also Figure S1 available online). Initially, we based these upon electroretinography (although see also Figure 3). As melanopsin is increasingly active at brighter backgrounds, our first control was to show that ERG responses to daylight and mel-low conditions (50 ms transition from "background" to "flash" spectra at 1 Hz) were indistinguishable at a moderate irradiance (Figures 1E and 1F). We next showed that responses to these stimuli were identical in mice lacking melanopsin (*Opn4*$^{-/-}$*;* *Opn1mw*^{R}) at both moderate (not shown) and high background irradiances (Figures 1G and 1H).

Melanopsin-Driven Modulation of the Cone Flash ERG

Having validated daylight and mel-low stimuli, we continued to present them to *Opn1mw*^{R} mice at a high, melanopsin-active irradiance. ERG b-wave amplitude was reproducibly enhanced in the mel-low condition (Figure 2B). This change built up gradually over several minutes following transition from mel-low to daylight backgrounds (Figure 2C) and was reversible (Figures 2D and 2E). By changing our spectra to produce flash stimuli representing a range of increases in effective cone photon flux, we described contrast-response relationships under background and mel-low conditions for this flash ERG. Behavioral contrast sensitivity has recently been reported to be impaired in *Opn4*$^{-/-}$ mice [27]; however, we did not find an equivalent effect of dynamic modulations in melanopsin activity. Thus, b-wave amplitude was greater across most contrasts in the mel-low condition, indicating increased response gain, but no change in contrast sensitivity per se (Figures 2F and 2G).

Melanopsin-Driven Changes in Visual Response Extend to the dLGN

We next recorded responses of neurons in the dLGN, which allowed us to determine whether these changes were

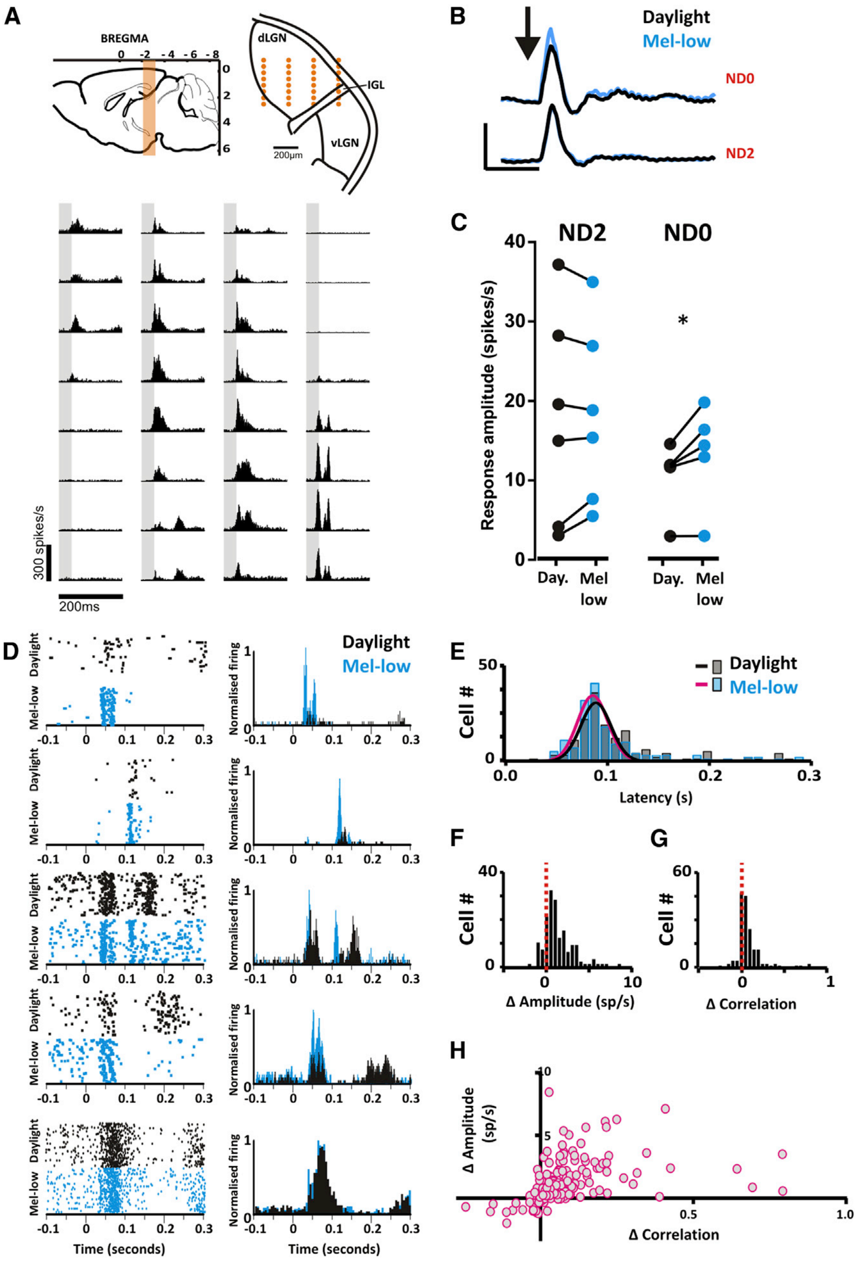

Figure 3. Melanopsin-Dependent Changes in the dLGN Response to Flash Stimuli

(A and B) Responses to daylight and mel-low flashes (50 ms flash at 1 Hz, 200 repeats) in contralateral dLGN were recorded using multielectrode probes. (A) Top: targeted region in sagittal (left) and coronal (right) views, with projected recording site positions superimposed in orange. Bottom: representative multiunit responses recorded in the dLGN of an *Opn1mw*^{R} mouse. Histograms show mean changes in firing rate detected at each of 32 recording sites (shown in gray). (B) Changes in multiunit activity averaged across multiple recording sites for a representative *Opn1mw*^{R} mouse in daylight and mel-low conditions (black and blue lines, respectively), at full (ND0) and 100 times reduced (ND2) irradiance. Scale bars, 250 ms (x); 10 spikes/s (y).

(C) Mean change in multiunit firing rate over the 200 ms following flash onset for recording sites across the dLGN of six *Opn1mw*^{R} mice was significantly different under daylight (black) and mel-low (blue) at ND0 but not ND2 (paired t tests). See also Figure S2D. Responses of representative single units reveal that the change in global response amplitude between mel-low and daylight conditions at high irradiance (ND0) are associated with alterations in response properties for many single units. At left are peri-event rasters for 200 repeats, with associated peri-event time histograms to the right (flash onset at time zero).

(D) Responses of representative single units reveal that the change in global response amplitude between mel-low and daylight conditions at high irradiance (ND0) are associated with alterations in response properties for many single units. At left are peri-event rasters for 200 repeats, with associated peri-event time histograms to the right (flash onset at time zero).

(E–H) The distribution of response latencies for single units (E) reveals a slight change in timing between conditions but no significant alteration in response synchrony between mel-low and daylight conditions ($p > 0.05$ for F-test comparison of Gaussians fitted to the two distributions, $R^2 > 0.8$). Distributions of differences in response amplitude (F) and trial-to-trial reproducibility (G; quantified as Pearson's correlation coefficient) for single units between conditions (mel-low − daylight) reveal that a large fraction of units showed increases in response amplitude and reliability in the mel-low condition. There was a significant correlation between these two features (Pearson's correlation coefficient $R^2 = 0.16$, $p < 0.0001$) across the population of units (H). Data shown are from 161 single units isolated in six mice.

propagated beyond the retina. Using multichannel electrodes, we recorded responses to daylight and mel-low flash paradigms across the contralateral dLGN (Figure 3A). To determine whether the change in ERG b-wave amplitude had a simple correlate in the dLGN response, we computed the mean change in firing of multiunit activity across the dLGN of each mouse. We found changes in this parameter across the experimental conditions very similar to those observed in the ERG. Thus, flash response amplitude was reduced for daylight versus mel-low in *Opn1mw*^{R} animals at high backgrounds, but not in any of the control conditions (Figures 3B and 3C; Figures S2A–S2C).

To explore the origins of this alteration in global response amplitude, we turned to examining responses at the single-unit level. Of 272 single units isolated from seven mice, 161 responded to the flash with, in all cases, increased firing. Many units showed qualitative differences in response to mel-low and daylight conditions (Figure 3D). In some cases, the temporal profile of stimulus evoked spikes changed (see Figure S3 for analysis of this feature). However, such changes in timing could not explain the change in global response amplitude (Figure 3B), as the predominant peak in firing was no less synchronous across the dLGN population in the daylight than mel-low condition (Figure 3E). Instead, we found that the increase in global response amplitude in mel-low could be attributed to a similar change at the single-unit level, with >75% of units showing greater light-evoked firing in this condition (Figure 3F). Surveying responses to individual trials (Figure 3D) indicated

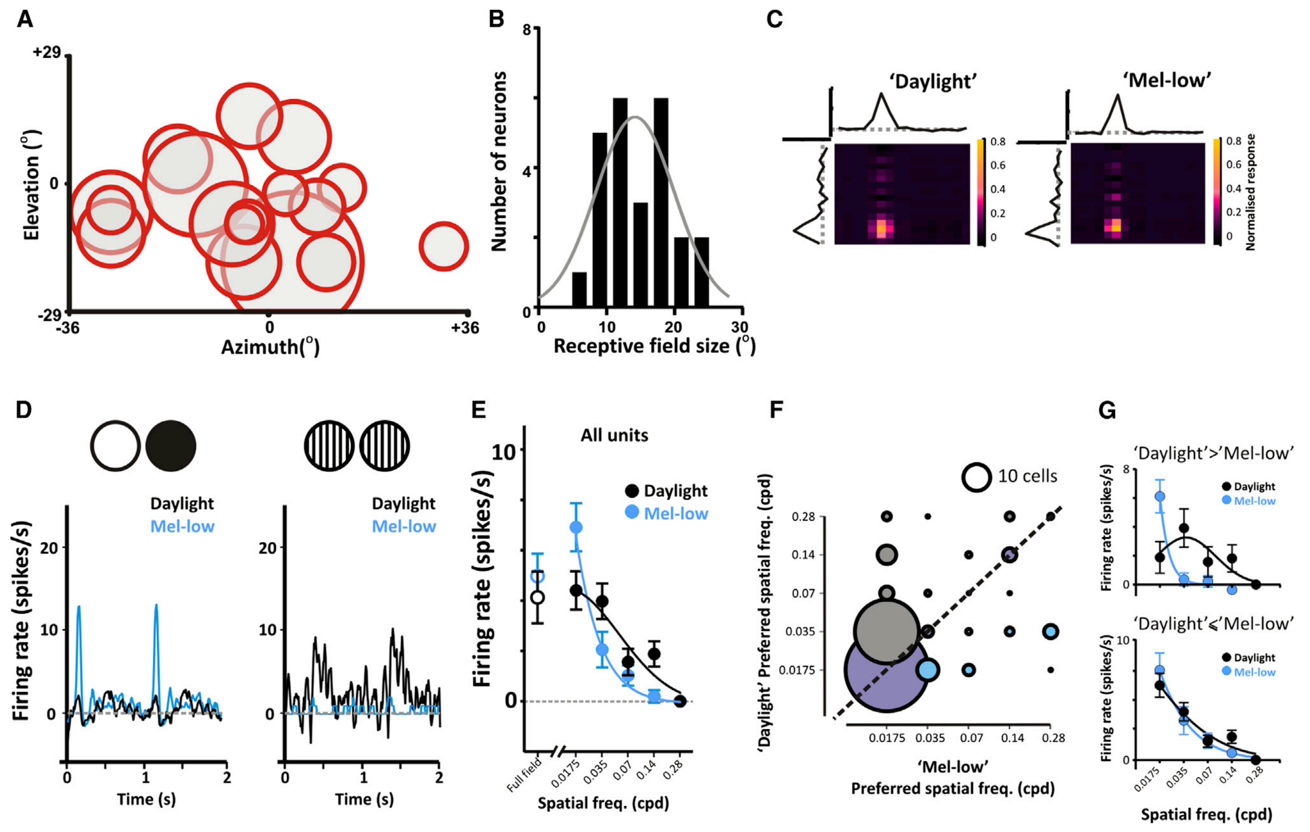

Figure 4. Spatial Tuning Properties Are Modulated According to Melanopsin Excitation

(A–C) Spatial receptive field mapping. RFs single dLGN units were mapped under both daylight and mel-low conditions using a white noise presentation of black-and-white horizontal and vertical bars upon a gray background. (A) The location of spatial RFs for 17 cells from 4 *Opn1mw*^{R} mice (circles show half-maximum width of Gaussian fits of spike-triggered averages in each dimension). (B) RF diameter of these units (half-maximum bandwidth of Gaussian) ranged from 5.6° to 23°. (C) Spatial RFs mapped under both daylight and mel-low conditions for a representative unit. Heatmaps (scale is normalized response) show response to bars in each orientation under each condition, derived spike triggered averages plotted above and to the left. Note the similarity in RF position in each condition.

(D–G) Many units that failed to respond to the full-field flash under the daylight spectrum responded well to spatially structured stimuli. (D) Mean peri-event histograms of baseline-subtracted firing rate to 200 presentations of full-field flashes (left) and 40 presentations of inverted gratings (0.035 cpd) in a representative unit under daylight (black) and mel-low (blue) conditions. Flashes occurred at time 0 and 1 and grating inversions every 0.5 s starting at zero. Spatial frequency tuning was examined by recording responses to 1 Hz inverting gratings at four orientations, at five different spatial frequencies (0.035–0.56 cpd) in mel-low and daylight conditions. (E) Mean modulation in firing rate for individual cells when the grating in preferred orientation revealed a shift in the spatial frequency tuning between mel-low and daylight condition. Responses to full-field stimuli plotted for reference (50 ms flashes at 1 Hz). (F) Distribution of preferred spatial frequency (maximum response) for single units in each condition; circle width scaled to represent number of cells in each group (circle size for 10 cells shown above for comparison). Blue circle show cells with higher preferred spatial frequency in mel-low; gray cells show higher preferred spatial frequency in daylight, and lilac cells with no change. (G) The mean response at different spatial frequencies from (E) is replotted for those cells showing a higher preferred spatial frequency in daylight (gray circles in F; top graph), or the remaining cells (lilac and blue circles in F; bottom graph). Data in (E) and (G) are fitted with partial Gaussian curves. Data shown indicate mean ± SEM.

that a predominant origin for this effect might be changes in trial-to-trial reproducibility. Indeed, there was an increase in response reproducibility mel-low (paired t test, $p < 0.0001$; Figure 3G) that correlated strongly with mean response amplitude (Pearson's correlation coefficient $R^2 = 0.16$, $p < 0.0001$; Figure 3H). These effects were absent in melanopsin knockout mice (Figures S2D and S2E).

Changes in Spatial Frequency Preference

A predominant effect of switching from mel-low to daylight conditions was thus a reduction in trial-to-trial reproducibility of flash responses. This result is perhaps counterintuitive, insofar as one might have expected visual performance to be improved under the daylight spectrum, which approximates the mouse's experience of natural conditions. A possible explanation for this apparent paradox is that the reduction in reproducibility of responses to the full-field flash reflects a change in preference toward more complex visual stimuli. To explore this possibility, we modified a digital mirror device projector (DLP LightCommander, Logic PD), replacing the intrinsic light engine with our own multispectral light source. This allowed us to render back-projected, structured images in the same mel-low versus daylight spectra.

We first looked at spatial response properties by mapping receptive fields (RFs) in each condition. As expected, the spatial location of RF centers was invariant between the two conditions (Figure 4A). As previously reported [28, 29], there was substantial diversity in RF size between units (Figure 4B. Nonetheless, we did not find a single example of a significant change in the size of RF centers between mel-low and daylight ($p > 0.05$ for F-test comparisons of Gaussians fitted to spike-triggered averages to bars presented in vertical and horizontal

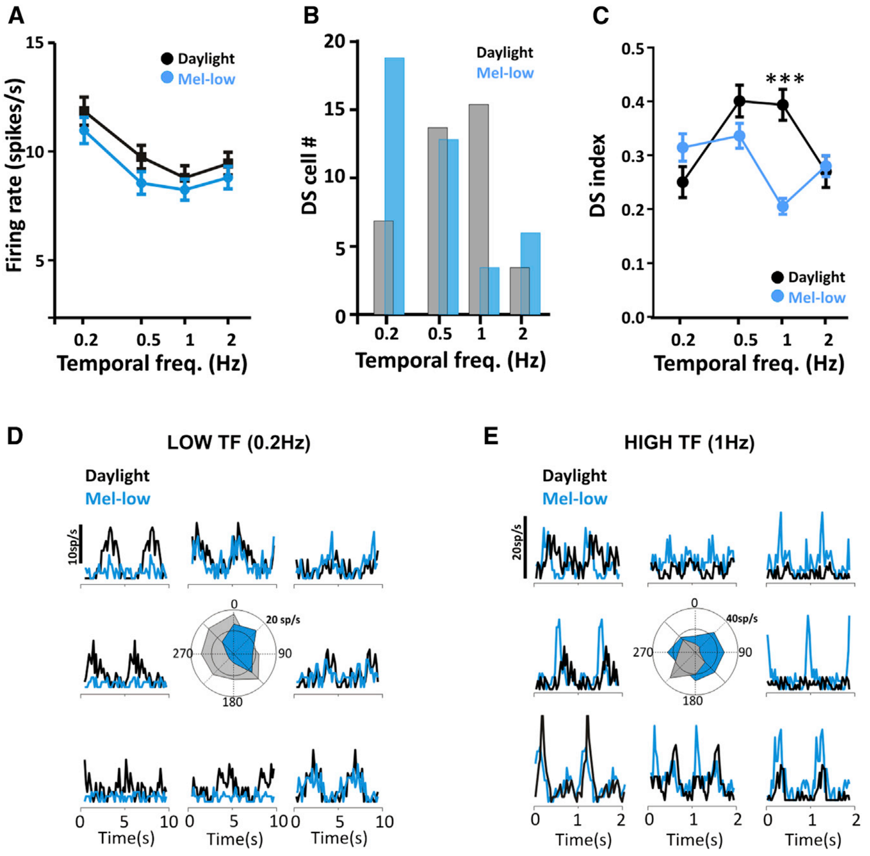

Figure 5. Changes in Temporal Frequency Tuning among Direction-Sensitive Units

(A) Temporal frequency tuning was assessed by recording responses to drifting gratings (0.035 cpd) moving in eight orientations, at four temporal frequencies (0.2–2 Hz) in both daylight and mel-low conditions. Across all temporal frequencies, response amplitude (mean ± SEM peak firing rate; for all light-responsive units, n = 117 from five mice) was higher in the daylight condition, but there was no change in the relative effectiveness of different frequencies between daylight and mel-low. Two-way repeated-measures ANOVA, significant effects of frequency ($p < 0.0001$) and condition ($p = 0.036$), but not of the frequency-condition interaction ($p > 0.05$).

(B–E) A subset of cells showed direction sensitivity at one or more temporal frequencies. (B) The number of direction-sensitive cells as a function of grating temporal frequency in daylight (gray) and mel-low (blue) conditions, revealing a switch to more abundant direction selectivity for higher temporal frequencies in the daylight condition. This was apparent also at the single-unit level, with examples of cells switching the temporal frequency at which they were most direction sensitive. (C) The DS index was compared for all cells that were classed as direction sensitive in any condition, at any frequency (data shown indicate mean ± SEM). This again revealed a significant change in the temporal frequency tuning of cell responses in mel-low and daylight conditions, with repeated-measures two-way ANOVA revealing significant increase in DS index at 1 Hz in the daylight condition ($p < 0.001$). (D and E) Representatives of such behavior, with peristimulus rate histograms and associated polar plots depicting response amplitude for movement in each of eight directions differing markedly under daylight (black) and mel-low (blue) conditions. The temporal frequency (TF) at which the grating was presented is provided above. (D) A cell with selectivity for direction of slow moving gratings (0.2 Hz) under the mel-low but not day-light condition. (E) A cell showing direction sensitivity at higher frequencies in daylight but not mel-low.

orientations). In common with reports for mice dLGN [28, 29], however, we were unable to map robust surround components of the RF (Figure 4C). Because of this, we used an additional approach to determine spatial feature preference and presented contrast inverting gratings over a range of spatial frequencies.

The inverting gratings revealed responses to a wide range of spatial frequencies in both conditions (Figures 4D and 4E). As a population, however, there was a tendency for larger-amplitude responses to finer gratings in the daylight condition. The origin of this effect was revealed by comparing the preferred spatial frequency of cells in mel-low and daylight (Figure 4F). Although many (42/112) cells showed maximal responses to the same spatial frequency in both conditions, there was an overall tendency for cells to prefer higher spatial frequencies in daylight compared to mel-low (Wilcoxon matched-pairs signed rank test, $p < 0.05$). Notably, those cells switching spatial frequency preference also had the largest reduction in full-field flash response (1.7 ± 1.2 versus 7.5 ± 1.6 spikes/s in daylight and mel-low, respectively; paired t test, $p < 0.001$) and accounted for all the change in spatial frequency tuning between conditions (Figure 4G). The most common effect (displayed by 51% of units changing) was a shift from preferring the lowest frequency (0.0175 cycles per degree [cpd]) to 0.035 cpd in the daylight condition. This optimal grating size, which equates to a visual angle of ~14°, is smaller than the calculated RF center of these cells, but would be predicted to provide good contrast between the RF center and neighboring points of visual space. Such behavior could be readily explained by strengthening of an inhibitory surround to the RF. In any event, these data show that many dLGN neurons adjust spatial frequency tuning between mel-low and daylight conditions. For a substantial fraction this entails a fundamental realignment from responding most to full-field stimuli to preferring spatial patterns. Together, these changes provide a simple explanation for the surprising reduction in responses to full-field flashes in the daylight condition.

Changes in Temporal Frequency Tuning

We next asked whether the changes in temporal profile of responses to full-field flashes observed upon switching from mel-low to daylight conditions (see Figures 3D and S3) could reflect alterations in temporal frequency preference of dLGN units and therefore examined responses to drifting gratings at different temporal frequencies (spatial frequency fixed at 0.035 cpd over eight directions of motion). More than 80% of cells responding to drifting gratings responded to movements in all directions. Response amplitude increased across all temporal frequencies under the daylight condition (Figure 5A), consistent with the view that responses to stimuli with appropriate spatial structure are improved in this condition (Figure 4). However, there was no difference in temporal frequency tuning under mel-low and daylight spectra. Nor could we discern any relationship between responses to the full-field flash and drifting gratings to explain the changes in the temporal profile of flash responses between these conditions (Figures S3C–S3E).

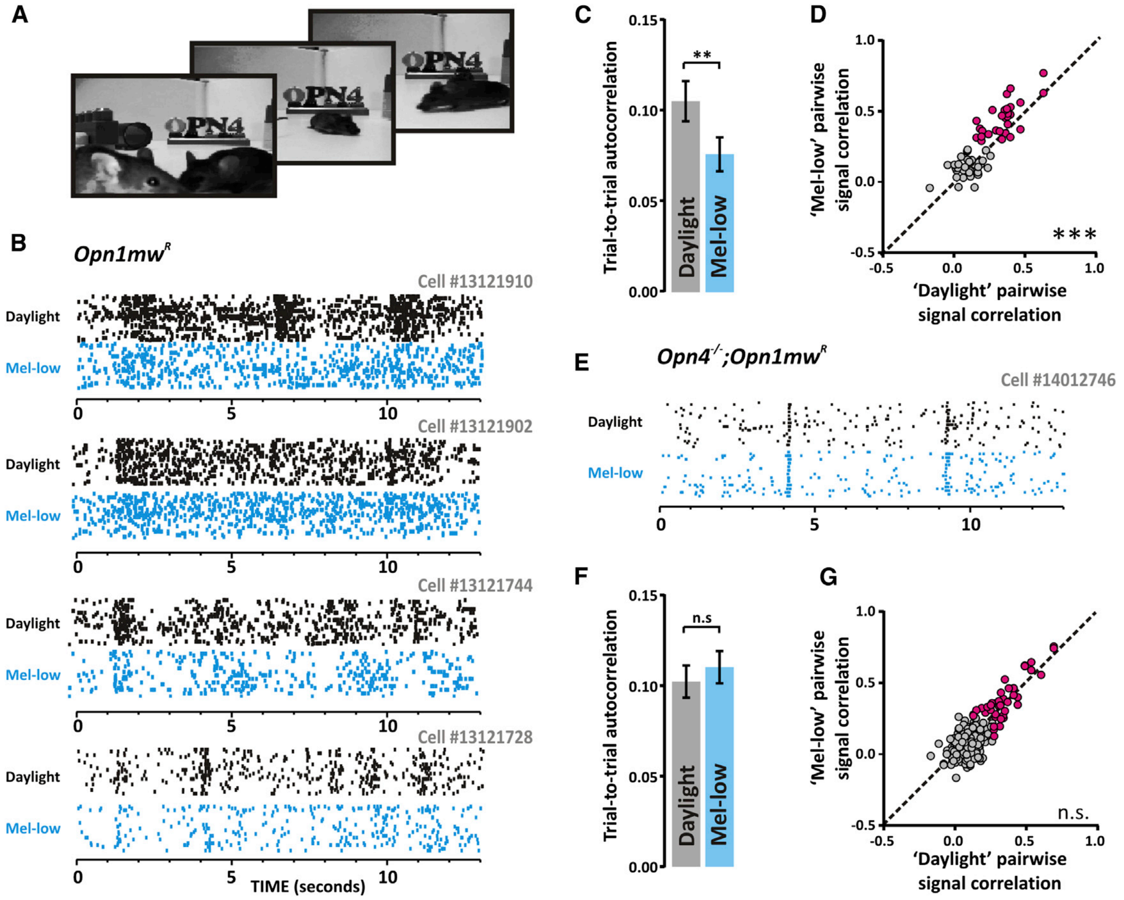

Figure 6. Responses to Natural Scenes in Daylight and Mel-Low Conditions

(A) Example frames from a short natural movie (mice moving around an open arena in horizontal view) presented to *Opn1mw*[R] and *Opn4*$^{-/-}$;*Opn1mw*[R] mice.

(B) Raster plots for four representative units from *Opn1mw*[R] mice exposed to 50 repeats of the movie under either daylight (black) or mel-low (blue) spectra.

(C) The trial-to-trial reproducibility of firing patterns of individual units (estimated by Pearson's correlation coefficient; autocorrelation) was significantly higher in the daylight condition (paired t test, $p < 0.01$; $n = 28$ from four *Opn1mw*[R] mice).

(D) The similarity in firing pattern between pairs of units from the same mouse under mel-low and daylight conditions is depicted as signal correlation (Pearson's correlation coefficient; 28 pairs from four *Opn1mw*[R] mice). Signal correlation was significantly reduced under the daylight spectrum (paired t test, $p < 0.001$). This was especially apparent for those pairs of units showing high correlation (>0.25) in either or both conditions, the pink data points.

(E–G) The response of melanopsin knockout mice to the movie was similar in daylight and mel-low spectra. (E) Raster plot of firing of a representative *Opn4*$^{-/-}$;*Opn1mw*[R] unit in response to the movie under daylight (black) and mel-low (blue) conditions. At the population level (33 single units from three mice), there was no difference in autocorrelation (F) or signal correlation (G) between daylight and mel-lo conditions. Data shown indicate mean ± SEM.

These drifting gratings did, however, reveal another change in feature selectivity between mel-low and daylight conditions. As previously reported [29], a subset of units preferred for movement in a particular direction (direction sensitive; see Experimental Procedures). Under both mel-low and daylight spectra, these direction-sensitive cells accounted for ~16% of the total. For all such cells, the degree of response suppression for motion in the null direction varied according to the temporal frequency of the stimulus (representative examples shown in Figures 5D and 5E). Under the mel-low condition the prevalence of direction sensitivity was highest for the slowest movement (0.2 Hz; corresponding to grating motion of 6°/s), while under daylight this occurred at 1 Hz (29°/s; Figures 5B–5D). This switch to preferring higher frequencies was also apparent in comparisons of direction selectivity (DS) index (Figure 5E). These data therefore indicate that, for at least a subset of direction-sensitive cells, the change in melanopsin effective photon flux drives an alteration in preferred speed of motion.

Melanopsin-Dependent Changes in Response to Natural Movies

Having described differences in feature preference under daylight versus mel-low spectra using artificial stimuli, we finally set out to determine their implication for the dLGN's ability to encode naturalistic stimuli. To this end, we projected movies of a natural scene (mice moving around an open arena; Figure 6A) in each condition. This short movie (30 s) was presented repeatedly over a 30 min period, enabling us to identify many units (~30% of all cells) with highly reproducible firing patterns across multiple presentations (see Supplemental Experimental Procedures, Natural Movie Correlation Analyses). We found many instances in which the firing pattern of units from *Opn1mw*[R] mice was reliable for multiple presentations of the movie under either mel-low or daylight spectra, but diverged substantially between the two conditions (Figure 6B). These were absent from recordings in *Opn4*$^{-/-}$;*Opn1mw*[R] animals (Figure 6E).

Based upon the improvement in trial-to-trial reproducibility of responses to artificial stimuli with appropriate spatial structure under the daylight spectrum (Figure 4C), we expected to see a similar increase in response reliability for the naturalistic movies in this condition. We therefore computed the trial-to-trial correlation for each unit under each condition ("autocorrelation"; see Experimental Procedures), and found that single-unit responses were indeed more reliable enhanced in the daylight condition (Figure 6C). Importantly, this effect was absent in *Opn4*$^{-/-}$*;Opn1mw*^{R} animals (Figure 6F).

A couple of features of the response to artificial stimuli suggest that the visual representation of visual scenes across dLGN neurons could be more diverse (richer) in the daylight condition: changes in spatial frequency tuning (Figure 4) indicate that a subset of dLGN neurons provide finer spatial resolution of visual features; and alterations in the behavior of direction-sensitive units (Figure 5) imply that this aspect of motion is tracked over a wider range of velocities. To determine whether responses across the population were indeed more varied in the daylight condition, we computed the correlation between mean firing response patterns of pairs of units from each animal ("signal correlation"). To limit the risk of sampling bias, we did not restrict our analysis to units with equivalent spatial RFs or related feature selectivity; our only constraint was that the units included had an RF centered within the projection screen and that they responded consistently to at least some aspect of the movie (see Experimental Procedures). As might be predicted, therefore, we found substantial variability in the signal correlation between pairs of units within a single animal. Nevertheless, even in this unfiltered data set there was a significant increase in signal correlation in the mel-low compared to daylight condition in *Opn1mw*^{R} animals (Figure 6D). This effect was enhanced if one restricted analysis to those pairs with relatively high pairwise correlations in either condition (Pearson's linear correlation coefficient > 0.25). Once again, *Opn4*$^{-/-}$*;Opn1mw*^{R} animals lacked this change (Figure 6G). We conclude that melanopsin-driven adjustments in the visual response allow the visual code to provide a richer, less redundant representation of natural scenes.

Discussion

Here, we have applied the concepts of metamerism and receptor silent substitution to compare responses to visual stimuli presented under conditions that differ only in their effective photon flux for melanopsin. The first condition (daylight) approximates the mouse's experience of natural daylight, while in the second condition (mel-low), those wavelengths to which melanopsin is most responsive, were selectively depleted. Switching between daylight and mel-low conditions resulted in substantial alterations in visual responses in the retina and dLGN. These were caused by fine changes in stimulus selectivity in spatial and temporal dimensions at the single-neuron level. Such changes in feature preference were associated with quantitative improvements in the dLGN's ability to encode natural scenes when presented in the daylight condition.

To reveal the impact of melanopsin on conventional vision, it was necessary to devise a method of selectively modulating the activity of melanopsin in an animal with a fully functional visual system. Our approach adopts the concept of metamerism: that stimuli differing markedly in spectral composition can appear indistinguishable for one or more classes of photoreceptor. In this case, our mel-low and daylight conditions are designed to appear equivalent for cones. One feature of the approach is that it cannot reveal any contribution of ipRGCs to adaptation at lower irradiances when their activity depends upon rods and cones. Our data thus likely underestimate the contribution of ipRGCs to retinal physiology.

While our conclusions do not require that the rate of photon capture by all cones is absolutely identical under mel-low and daylight conditions (a practical impossibility), it is important that they are sufficiently similar as to make any difference in cone response fall below the detection limits of our methods. We are confident that this is the case. We have designed these stimuli based upon extensive measurements of the in vivo spectral sensitivity of mouse cones [25, 30, 31]. Moreover, both ERG and dLGN responses are indistinguishable between mel-low and daylight conditions when working at light levels below those favored by melanopsin and under all conditions in mice lacking melanopsin. Finally, the changes in response properties we observe build up over several minutes of exposure to the new background (Figure 2), consistent with a gradual, melanopsin-dependent adjustment in visual response, but not with a fundamental difference in the cone experience of the two conditions, which should be apparent from the very first presentation of the new stimulus.

What functional advantage could be gained by adjusting vision according to a melanopsin-dependent assessment of irradiance? Light adaptation in visual circuits involves changes in the behavior of individual synapses and the nature and extent of connections between pairs of neurons. One function of this adaptation is to conserve the visual code against changes in irradiance; another is to adjust circuitry to take advantage of irradiance-dependent increases in signal reliability and/or changes in photoreceptor temporal resolution [1, 2, 32–34]. However, these two features are in some ways contradictory. Viewed from the perspective of a circuit element charged with achieving the second of these goals, the fact that light adaptation upstream in the circuit minimizes the impact of changes in background light levels on incoming signals makes it more difficult to accurately assess irradiance. Basing adaptation instead on an independent measure of irradiance not subject to the same light adaptation processes, as shown here for melanopsin, is one solution to this problem. If such a general mechanism were to explain the influence of melanopsin in the mouse retina, one might expect to find independent irradiance codes also in other visual systems. Indeed, this seems to be the case. Thus, in fish and other lower vertebrates, a range of non-rod, non-cone photopigments are expressed widely in inner retinal neurons [35–38]. Meanwhile, in *Drosophila*, a recent publication reports regulation of vision by cryptochrome, the fly's version of an irradiance measurement system [39].

The simplest effect we observe is smaller responses to a full-field flash in the daylight condition. This is apparent in the ERG and in the LGN at both population and single-unit levels. Using spatially structured stimuli reveals that this effect represents a widespread shift in spatial frequency tuning. Many dLGN units that respond well to full-field flashes and gratings at the lowest spatial frequencies in mel-low switch to preferring higher spatial frequencies under daylight ($\sim$0.035 cpd). Future work will be required to define the changes in visual circuits underlying these effects. There are multiple reports of luminance- and/or irradiance-dependent changes in spatial frequency preference (although generally at lower light levels than

studied here [1, 2, 40–42]). Where elucidated, mechanisms for such changes involve alterations in the inhibitory surround provided by retinal horizontal and amacrine cells. These cell types therefore seem likely targets of melanopsin control. Inhibitory amacrine cells are also responsible for establishing direction selectivity [43, 44], implicating changes in their activity also as a likely origin of the melanopsin-dependent change in temporal frequency tuning of direction sensitive units that we observe (Figure 5).

While our ERG data reveal that at least some melanopsin-driven changes in visual response originate with the retina, our study does not preclude a contribution of central processing within the brain. These retinal changes could themselves involve feedback via centrifugal histaminergic [45, 46] and/or serotonergic [47] projections. Moreover, thalamic and thalamocortical circuits allow plenty of opportunity for fine-tuning visual responses. It may therefore be that some of the changes in visual feature selectivity we see in the dLGN reflect an impact of melanopsin-based assessments of irradiance within the brain itself. These could extend to melanopsin-driven increases in arousal or attention, although any such effect would likely be specific to vision as responses to another sensory stimulus were indistinguishable under the two lighting conditions (Figure S4).

Our findings add to the growing evidence that the sensory requirements of ipRGCs should influence lighting design [48]. Most incandescent and fluorescent lighting is long wavelength biased compared to natural light and thus deficient in those wavelengths to which melanopsin is most sensitive, effectively recapitulating our mel-low condition. While the degree to which melanopsin excitation is reduced in our experimental mel-low condition is probably several times greater than in most artificial lighting, these data do imply that choosing light sources that more closely approximate melanopsin's experience of daylight may bring qualitative improvements in visual performance.

Experimental Procedures

In Vivo Electrophysiology

Animal care was in accordance with the UK Animals, Scientific Procedures, Act (1986). ERGs and dLGN responses were recorded concurrently from 13 *Opn1mw*^{R} and eight *Opn4*$^{-/-}$;*Opn1mw*^{R} male mice (aged 3–6 months) anesthetized with urethane (1.6 g/kg, 30% w/v; Sigma-Aldrich). dLGN recordings employed a 32-channel probe (A4x8-5mm-50-200-413; Neuronexus). Recording methods as previously reported [49]. In addition, a separate set of mice (six *Opn1mw*^{R} and three *Opn4*$^{-/-}$; *Opn1mw*^{R} males) was used to record responses in the dLGN to spatially structured stimuli.

Visual Stimuli

Full-field visual stimuli were generated using three independently controlled LEDs (λ_{max} 365, 460, and 600 nm; Cairn Research). LEDs were combined to generate two background and stimulus combinations that are summarized in Figure 1D. The approach is equivalent to that described in [48], using spectral efficiency functions available at http://lucasgroup.lab.ls.manchester.ac.uk/research/measuringmelanopicilluminance. Structured images were presented using a custom-made light source containing four independently controlled LEDs (λ_{max} 405, 455, 525, and 630 nm; Phlatlight PT-120 Series, Luminus Devices), directed into a digital mirror device projector (DLP LightCommander).

Supplemental Information

Supplemental Information includes Supplemental Experimental Procedures and four figures and can be found with this article online at http://dx.doi.org/10.1016/j.cub.2014.09.015.

Acknowledgments

This work was supported by grants from the European Research Council and the Biological Sciences and Biotechnology Research Council to R.J.L. We thank Jonathan Wynne for technical assistance.

Received: June 12, 2014
Revised: August 6, 2014
Accepted: September 3, 2014
Published: October 9, 2014

References

1. Barlow, H.B., Fitzhugh, R., and Kuffler, S.W. (1957). Change of organization in the receptive fields of the cat's retina during dark adaptation. J. Physiol. *137*, 338–354.
2. Enroth-Cugell, C., and Lennie, P. (1975). The control of retinal ganglion cell discharge by receptive field surrounds. J. Physiol. *247*, 551–578.
3. Bloomfield, S.A., and Völgyi, B. (2009). The diverse functional roles and regulation of neuronal gap junctions in the retina. Nat. Rev. Neurosci. *10*, 495–506.
4. Witkovsky, P. (2004). Dopamine and retinal function. Doc. Ophthalmol. *108*, 17–40.
5. Warren, E.J., Allen, C.N., Brown, R.L., and Robinson, D.W. (2003). Intrinsic light responses of retinal ganglion cells projecting to the circadian system. Eur. J. Neurosci. *17*, 1727–1735.
6. Dacey, D.M., Liao, H.W., Peterson, B.B., Robinson, F.R., Smith, V.C., Pokorny, J., Yau, K.W., and Gamlin, P.D. (2005). Melanopsin-expressing ganglion cells in primate retina signal colour and irradiance and project to the LGN. Nature *433*, 749–754.
7. Tu, D.C., Zhang, D., Demas, J., Slutsky, E.B., Provencio, I., Holy, T.E., and Van Gelder, R.N. (2005). Physiologic diversity and development of intrinsically photosensitive retinal ganglion cells. Neuron *48*, 987–999.
8. Brown, T.M., Gias, C., Hatori, M., Keding, S.R., Semo, M., Coffey, P.J., Gigg, J., Piggins, H.D., Panda, S., and Lucas, R.J. (2010). Melanopsin contributions to irradiance coding in the thalamo-cortical visual system. PLoS Biol. *8*, e1000558.
9. Berson, D.M., Dunn, F.A., and Takao, M. (2002). Phototransduction by retinal ganglion cells that set the circadian clock. Science *295*, 1070–1073.
10. Hattar, S., Liao, H.W., Takao, M., Berson, D.M., and Yau, K.W. (2002). Melanopsin-containing retinal ganglion cells: architecture, projections, and intrinsic photosensitivity. Science *295*, 1065–1070.
11. Provencio, I., Rollag, M.D., and Castrucci, A.M. (2002). Photoreceptive net in the mammalian retina. This mesh of cells may explain how some blind mice can still tell day from night. Nature *415*, 493.
12. Hattar, S., Lucas, R.J., Mrosovsky, N., Thompson, S., Douglas, R.H., Hankins, M.W., Lem, J., Biel, M., Hofmann, F., Foster, R.G., and Yau, K.W. (2003). Melanopsin and rod-cone photoreceptive systems account for all major accessory visual functions in mice. Nature *424*, 76–81.
13. Panda, S., Provencio, I., Tu, D.C., Pires, S.S., Rollag, M.D., Castrucci, A.M., Pletcher, M.T., Sato, T.K., Wiltshire, T., Andahazy, M., et al. (2003). Melanopsin is required for non-image-forming photic responses in blind mice. Science *301*, 525–527.
14. Hankins, M.W., and Lucas, R.J. (2002). The primary visual pathway in humans is regulated according to long-term light exposure through the action of a nonclassical photopigment. Curr. Biol. *12*, 191–198.
15. Sekaran, S., Foster, R.G., Lucas, R.J., and Hankins, M.W. (2003). Calcium imaging reveals a network of intrinsically light-sensitive inner-retinal neurons. Curr. Biol. *13*, 1290–1298.
16. Müller, L.P., Do, M.T., Yau, K.W., He, S., and Baldridge, W.H. (2010). Tracer coupling of intrinsically photosensitive retinal ganglion cells to amacrine cells in the mouse retina. J. Comp. Neurol. *518*, 4813–4824.
17. Joo, H.R., Peterson, B.B., Dacey, D.M., Hattar, S., and Chen, S.K. (2013). Recurrent axon collaterals of intrinsically photosensitive retinal ganglion cells. Vis. Neurosci. *30*, 175–182.
18. Zhang, D.Q., Wong, K.Y., Sollars, P.J., Berson, D.M., Pickard, G.E., and McMahon, D.G. (2008). Intraretinal signaling by ganglion cell photoreceptors to dopaminergic amacrine neurons. Proc. Natl. Acad. Sci. USA *105*, 14181–14186.
19. Bailes, H.J., and Lucas, R.J. (2010). Melanopsin and inner retinal photoreception. Cell. Mol. Life Sci. *67*, 99–111.
20. Do, M.T., and Yau, K.W. (2010). Intrinsically photosensitive retinal ganglion cells. Physiol. Rev. *90*, 1547–1581.

21. Barnard, A.R., Hattar, S., Hankins, M.W., and Lucas, R.J. (2006). Melanopsin regulates visual processing in the mouse retina. Curr. Biol. *16*, 389–395.
22. Dkhissi-Benyahya, O., Coutanson, C., Knoblauch, K., Lahouaoui, H., Leviel, V., Rey, C., Bennis, M., and Cooper, H.M. (2013). The absence of melanopsin alters retinal clock function and dopamine regulation by light. Cell. Mol. Life Sci. *70*, 3435–3447.
23. Rao, S., Chun, C., Fan, J., Kofron, J.M., Yang, M.B., Hegde, R.S., Ferrara, N., Copenhagen, D.R., and Lang, R.A. (2013). A direct and melanopsin-dependent fetal light response regulates mouse eye development. Nature *494*, 243–246.
24. Renna, J.M., Weng, S., and Berson, D.M. (2011). Light acts through melanopsin to alter retinal waves and segregation of retinogeniculate afferents. Nat. Neurosci. *14*, 827–829.
25. Smallwood, P.M., Olveczky, B.P., Williams, G.L., Jacobs, G.H., Reese, B.E., Meister, M., and Nathans, J. (2003). Genetically engineered mice with an additional class of cone photoreceptors: implications for the evolution of color vision. Proc. Natl. Acad. Sci. USA *100*, 11706–11711.
26. Szél, A., Röhlich, P., Caffé, A.R., and van Veen, T. (1996). Distribution of cone photoreceptors in the mammalian retina. Microsc. Res. Tech. *35*, 445–462.
27. Schmidt, T.M., Alam, N.M., Chen, S., Kofuji, P., Li, W., Prusky, G.T., and Hattar, S. (2014). A role for melanopsin in alpha retinal ganglion cells and contrast detection. Neuron *82*, 781–788.
28. Grubb, M.S., and Thompson, I.D. (2003). Quantitative characterization of visual response properties in the mouse dorsal lateral geniculate nucleus. J. Neurophysiol. *90*, 3594–3607.
29. Piscopo, D.M., El-Danaf, R.N., Huberman, A.D., and Niell, C.M. (2013). Diverse visual features encoded in mouse lateral geniculate nucleus. J. Neurosci. *33*, 4642–4656.
30. Jacobs, G.H., and Williams, G.A. (2007). Contributions of the mouse UV photopigment to the ERG and to vision. Doc. Ophthalmol. *115*, 137–144.
31. Jacobs, G.H., Williams, G.A., Cahill, H., and Nathans, J. (2007). Emergence of novel color vision in mice engineered to express a human cone photopigment. Science *315*, 1723–1725.
32. De Valois, R.L., Morgan, H., and Snodderly, D.M. (1974). Psychophysical studies of monkey vision. 3. Spatial luminance contrast sensitivity tests of macaque and human observers. Vision Res. *14*, 75–81.
33. Pasternak, T., and Merigan, W.H. (1981). The luminance dependence of spatial vision in the cat. Vision Res. *21*, 1333–1339.
34. Rieke, F., and Rudd, M.E. (2009). The challenges natural images pose for visual adaptation. Neuron *64*, 605–616.
35. Cheng, N., Tsunenari, T., and Yau, K.W. (2009). Intrinsic light response of retinal horizontal cells of teleosts. Nature *460*, 899–903.
36. Davies, W.I., Zheng, L., Hughes, S., Tamai, T.K., Turton, M., Halford, S., Foster, R.G., Whitmore, D., and Hankins, M.W. (2011). Functional diversity of melanopsins and their global expression in the teleost retina. Cell. Mol. Life Sci. *68*, 4115–4132.
37. Guido, M.E., Garbarino-Pico, E., Contin, M.A., Valdez, D.J., Nieto, P.S., Verra, D.M., Acosta-Rodriguez, V.A., de Zavalía, N., and Rosenstein, R.E. (2010). Inner retinal circadian clocks and non-visual photoreceptors: novel players in the circadian system. Prog. Neurobiol. *92*, 484–504.
38. Matos-Cruz, V., Blasic, J., Nickle, B., Robinson, P.R., Hattar, S., and Halpern, M.E. (2011). Unexpected diversity and photoperiod dependence of the zebrafish melanopsin system. PLoS ONE *6*, e25111.
39. Mazzotta, G., Rossi, A., Leonardi, E., Mason, M., Bertolucci, C., Caccin, L., Spolaore, B., Martin, A.J., Schlichting, M., Grebler, R., et al. (2013). Fly cryptochrome and the visual system. Proc. Natl. Acad. Sci. USA *110*, 6163–6168.
40. Farrow, K., Teixeira, M., Szikra, T., Viney, T.J., Balint, K., Yonehara, K., and Roska, B. (2013). Ambient illumination toggles a neuronal circuit switch in the retina and visual perception at cone threshold. Neuron *78*, 325–338.
41. Sagdullaev, B.T., and McCall, M.A. (2005). Stimulus size and intensity alter fundamental receptive-field properties of mouse retinal ganglion cells in vivo. Vis. Neurosci. *22*, 649–659.
42. Bisti, S., Clement, R., Maffei, L., and Mecacci, L. (1977). Spatial frequency and orientation tuning curves of visual neurones in the cat: effects of mean luminance. Exp. Brain Res. *27*, 335–345.
43. Fried, S.I., Münch, T.A., and Werblin, F.S. (2002). Mechanisms and circuitry underlying directional selectivity in the retina. Nature *420*, 411–414.
44. Yonehara, K., Balint, K., Noda, M., Nagel, G., Bamberg, E., and Roska, B. (2011). Spatially asymmetric reorganization of inhibition establishes a motion-sensitive circuit. Nature *469*, 407–410.
45. Akimov, N.P., Marshak, D.W., Frishman, L.J., Glickman, R.D., and Yusupov, R.G. (2010). Histamine reduces flash sensitivity of on ganglion cells in the primate retina. Invest. Ophthalmol. Vis. Sci. *51*, 3825–3834.
46. Frazão, R., McMahon, D.G., Schunack, W., Datta, P., Heidelberger, R., and Marshak, D.W. (2011). Histamine elevates free intracellular calcium in mouse retinal dopaminergic cells via H1-receptors. Invest. Ophthalmol. Vis. Sci. *52*, 3083–3088.
47. Repérant, J., Araneda, S., Miceli, D., Medina, M., and Rio, J.P. (2000). Serotonergic retinopetal projections from the dorsal raphe nucleus in the mouse demonstrated by combined [(3)H] 5-HT retrograde tracing and immunolabeling of endogenous 5-HT. Brain Res. *878*, 213–217.
48. Lucas, R.J., Peirson, S.N., Berson, D.M., Brown, T.M., Cooper, H.M., Czeisler, C.A., Figueiro, M.G., Gamlin, P.D., Lockley, S.W., O'Hagan, J.B., et al. (2014). Measuring and using light in the melanopsin age. Trends Neurosci. *37*, 1–9.
49. Allen, A.E., Cameron, M.A., Brown, T.M., Vugler, A.A., and Lucas, R.J. (2010). Visual responses in mice lacking critical components of all known retinal phototransduction cascades. PLoS ONE *5*, e15063.

Current Biology, Volume *24*

Supplemental Information

Melanopsin-Driven Light Adaptation in Mouse Vision

Annette E. Allen, Riccardo Storchi, Franck P. Martial, Rasmus S. Petersen, Marcelo A. Montemurro, Timothy M. Brown, and Robert J. Lucas

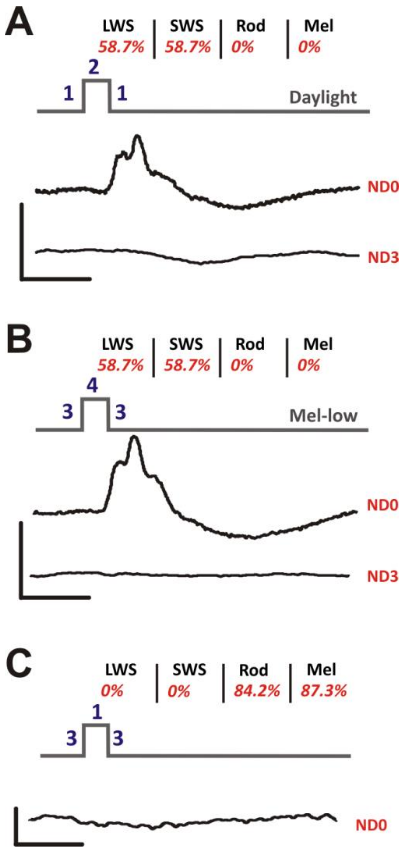

Figure S1. Further validation of spectra used to generate ‘daylight’ and ‘mel-low’ flash stimuli. a&b.) Relating to Figure 1. The flash stimuli presented under ódaylightô and ómel-lowô conditions were designed to be visible to cone but not rod (or melanopsin) photoreceptors. As a first confirmation that this was the case, we recorded ERGs at a background light intensity (ND3) 1000 fold lower than that used to generate the data presented in Figs 2 and 3 (ND0). Rods should be more, and cones, less active at this lower irradiance and we found that neither ódaylightô nor ómel-lowô stimuli evoked a measurable ERG under these conditions. Representative responses from a single animal are plotted in a&b. **c.)** The spectra used to generate background illumination under ódaylightô and ómel-lowô conditions (spectra 1 and 3 in Fig. 1 respectively) were designed to be isoluminant for cones. To determine whether this was the case, we attempted to record ERGs to transitions between the two spectra (a 50ms flash of spectrum 1 presented against a background of spectrum 3, at 1Hz; 200 repeats). This stimulus failed to elicit a measurable ERG at the irradiances used for these experiments in all 7 mice tested, consistent with the prediction that they are cone isoluminant. C shows a representative response from a single animal. Flash stimuli are shown in cartoon form above the ERG traces with the spectrum used to produce each element of the stimulus shown in blue (see Fig. 1 for spectral power distributions). Predicted Michelson contrasts for transitions between the stimulus pairs for each photoreceptor are given in red text above.

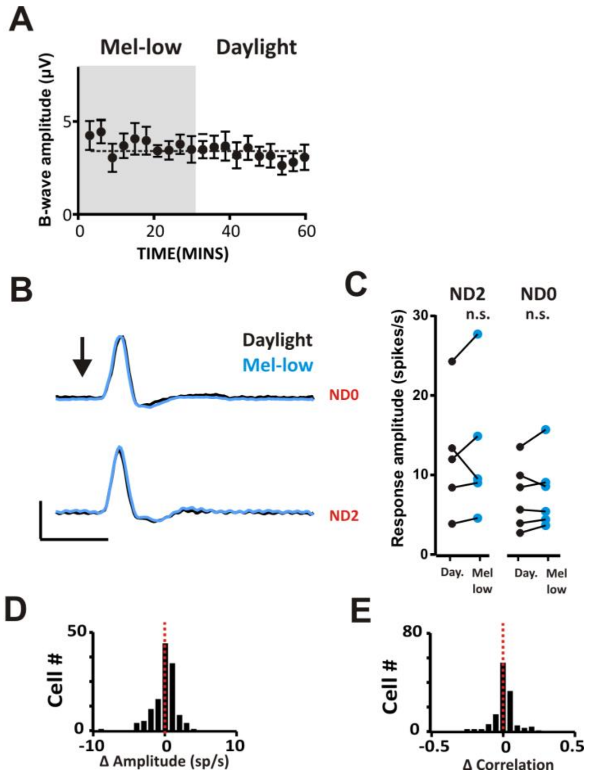

Figure S2. Retinal and dLGN responses to 'daylight' and 'mel-low' flash stimuli are indistinguishable in melanopsin knockout mice. Relating to Figures 2&3. **a.)** ERG response amplitudes recorded in 8 *Opn4*$^{-/-}$*; Opn1mw*^{R} mice exposed to repeated flashes across the transition from ómel-lowôto ódaylightôconditions; graph shows mean±SEM. These data were collected in parallel with those for *Opn1mw*^{R} data shown in Fig. 2c and compared by two-way ANOVA which revealed significant effects of genotype and genotype/time interactions ($p<0.05$); post-hoc Bonferonni multiple comparisons tests against data for that genotype at time zero revealed significant differences for many of the ódaylightôrecordings in *Opn1mw*^{R} mice (Fig. 2c) but not for any time point in melanopsin knockouts. **b.)** The change in multi-unit firing across recording sites in the dLGN of a representative *Opn4*$^{-/-}$*; Opn1mw*^{R} mouse in response to ódaylightô(black line) and ómel-lowô(blue) stimuli at ND0 and ND2. Scale bars: x=250ms, y =10spikes/s; stimuli as for Fig. 4; arrow depicts time of flash. **c.)** The mean change in multiunit firing rate in the 200ms following flash across recording sites in the dLGN of *Opn4*$^{-/-}$*; Opn1mw*^{R} mice was not significantly different between ódaylight (black symbols) and ómel-lowô (blue) at either ND0 or ND2 ($p>0.05$ paired t-tests. **d.)** The distribution of differences in this measure of response amplitude between conditions (ómel-lowôresponse ï ódaylightôresponse) for single units from *Opn4*$^{-/-}$*; Opn1mw*^{R} mice fails to recapitulate the shift in this parameter observed for *Opn1mw*^{R} mice (Fig. 3f). **e.)** The distribution of difference in autocorrelation (Pearsonõs correlation coefficient) in the response of single units between conditions (ómel-lowôï ódaylightõ) also shows that these melanopsin knockouts lack the change in this parameter reported for *Opn4*$^{-/-}$*; Opn1mw*^{R} mice (Fig. 3g).

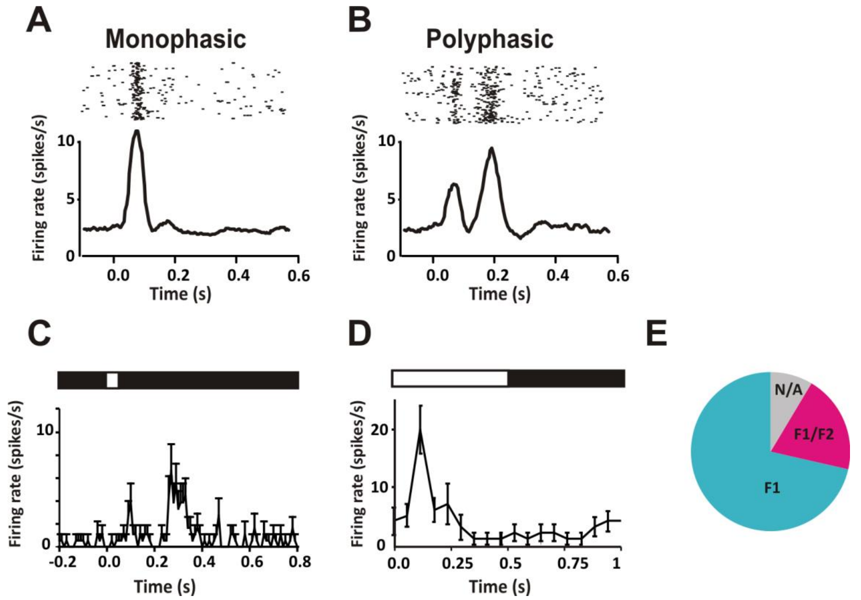

Figure S3. Exploring temporal changes in flash response profiles. Relating to Figures 3&4. There was no change in response latency between conditions, with the initial peak in firing lagging the flash by 89.1 ± 4.4msec in ómel-lowô and 81.8 ± 5.28msec in ódaylightô (mean±SEM; paired two-way T-test p=0.14). However, under either condition responses could be regarded as falling into two broad categories, in the first (termed ómonophasicô) there was a single peak in firing around 50ms after the flash (**a**), in the second (ópolyphasicô) this first peak was followed by one or more additional peaks over the 200ms post flash period (**b**). Above are peri-event rasters for 200 stimulus repeats, with associated peri-event time histograms below (flash onset at time 0). The two categories were sufficiently distinct to allow them to be defined by subjective assessments, but we confirmed the distinction using principle component analysis. Under any single background lighting condition the response of individual units did not change, but it was common to see units switch categories between ódaylightô and ómel-lowô conditions (Fig. 3d). In *Opn1mw*^{R} mice there was a net shift towards polyphasic responses when switching to the ódaylightô spectrum with 19% of all light responsive cells changing from monophasic to polyphasic. This effect was absent in *Opn4*$^{-/-}$*; Opn1mw*^{R} mouse confirming that it was driven by melanopsin. We asked whether the polyphasic response type was indicative of óON-OFFô response types by examining their responses to inverting gratings. **c&d** show representative responses of a polyphasic cell to the ódaylightô flash (**c**) and to inverting gratings (**d**) under ódaylightô conditions at 0.018cpd (stimulus presentation and data acquisition as described for the data in Figs 3 and 4). Note that when presented with the inverted grating this unit responds only to the increase in luminance over its receptive field centre indicating that it is an óONô cell. If there were a preponderance of ON-OFF responses for polyphasic units, one would expect to see frequency doubled responses to the inverted gratings (i.e. units responding to the appearance of white and black bars). To determine whether this was the case, the amplitude of dominant first and second harmonics (F1 and F2, corresponding to the 2Hz reversal frequency of the grating and 4Hz frequency doubling) were extracted from Fourier transforms of single unit responses to inverting gratings in the preferred orientation and phase. F1 and F2 were designated as significant if they lay outside 2 standard deviations of the Fourier transform of a shuffled version of the response. **e.)** A pie chart of the number of polyphasic units showing F1, both F1 and F2, no significant response (N/A) to the inverted

gratings indicates that most units designated as polyphasic lack the F2 component expected of óON-OFFô cells. Polyphasic cells did not differ in either preferred temporal or spatial frequency, nor were they more or less likely to be direction sensitive. Thus, while there clearly were changes in temporal profile of flash responses between conditions, and while these provide further evidence of melanopsin-dependent adjustments in the visual code, we cannot at present determine what significance this event has for encoding more natural visual stimuli.

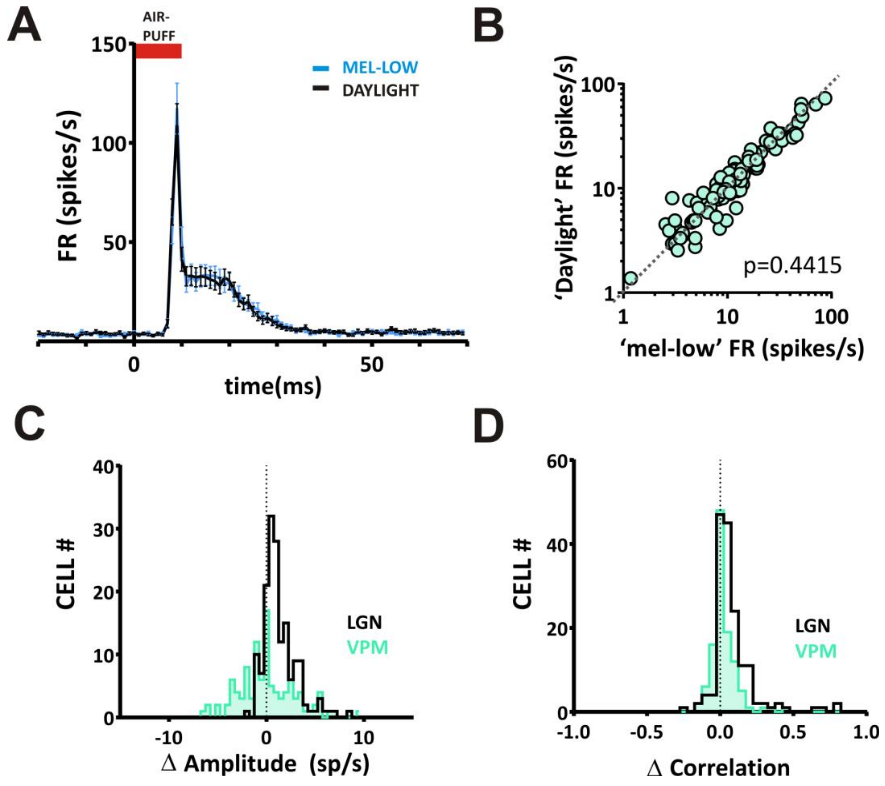

Figure S4. Changes in response in 'daylight' and 'mel-low' condition are specific to visual thalamus, and do not reflect a non-specific change in neuronal activity. Relating to Figures 4-6. **a&b.)** The amplitude of responses evoked by whisker movement (1Hz 10ms rostro-caudal air-puff stimulus), recorded in the ventral posteromedial region of the somatosensory thalamus (VPM), were also assessed in the ómel-lowô and ódaylightô conditions. No change in the response amplitude was detectable. **a,** the mean ±SEM firing rate of 112 units, recorded in 7 *Opn1mw*^{R} mice in each condition. **b,** a scatter plot of the response amplitude (change in FR in first 20ms of response) in each condition; paired T-test finds no change in amplitude between conditions (p=0.4415). **c&d,** A direct comparison of the distribution of the change in amplitude (**c**) and correlation (**d**), between ómel-lowô and ódaylightô conditions, in cells recorded in the dLGN (black; re-plotted from Fig.4) and VPM (green). Note that cells recorded in VPM are unaffected by the change in background, unlike those from dLGN.

SUPPLEMENTAL EXPERIMENTAL PROCEDURES

Electroretinography

ERGs were recorded from thirteen *Opn1mw*^{R} and eight *Opn4*$^{-/-}$*; Opn1mw*^{R} male mice (aged 3-6 months). Mice were from a mixed C57/BL6; 127sv strain. *Opn1mw*^{R} refers to the transgenic allele originally generated by Smallwood *et al*. (2003), and termed ñRò by them [S1]. *Opn4*$^{-/-}$ mice contain an insertion of tau-lacZ into the melanopsin gene locus, rendering mice ómelanopsin-knockoutô [S2]. All animal care was in accordance with the Animals, Scientific Procedures, Act of 1986 (UK). Animals were kept in a 12-hour dark/light cycle at a temperature of 22°C with food and water available *ad libitum*.

Anaesthesia was induced with an intra-peritoneal injection of urethane (1.6g/kg; 30%w/v; Sigma-Aldrich). A topical mydriatic (tropicamide, 1%; Chauvin Pharmaceuticals) and mineral oil (Sigma-Aldrich) were applied to the recording eye prior to placement of a corneal contact-lens type electrode. Mice were placed into a stereotaxic frame to keep a fixed head-position; a bite bar was also used for head support, and acted as a ground. A needle reference electrode (Ambu, Neuroline) was inserted approximately 5mm from the base of the contralateral eye. Electrodes were connected to a Windows PC via a signal conditioner (Model 1902 Mark III, CED) that differentially amplified (X3000) and filtered (band-pass filter cut off 0.5 to 200Hz) the signal, and a digitizer (Model 1401, CED). Core body temperature was maintained at 37°C throughout recording with a homeothermic heat mat (Harvard Apparatus). B-wave amplitudes were measured relative to baseline values (time of flash onset), since a-waves were not readily measurable

In vivo physiology

Neuronal activity within the dLGN to the flash stimuli was recorded concurrently with ERGs. In addition, a separate set of mice (six *Opn1mw*^{R} and three *Opn4*$^{-/-}$*; Opn1mw*^{R} male mice, aged 3-6 months) was used to record responses to spatially structure stimuli. After placement into the stereotaxic frame, the mouse's skull surface was exposed and a small hole drilled ~2.3mm posterior and ~2.3mm lateral to the bregma. A recording probe (A4x8-5mm-50-200-413; Neuronexus) consisting of 4 shanks spaced 200µm apart, each with 8 recording sites (spaced 50µm, sized 413µm^{2}), was lowered a depth of ~2.5-3mm into the brain, targeting the dorsal lateral geniculate nucleus (dLGN). In addition, in 7 *Opn1mw*^{R} mice, recordings were made in the ventral posteromedial region of the somatosensory thalamus (VPM; -1.8 - 2.1mm posterior and 1.4mm lateral to bregma; lowered ~3.5mm). Neural signals were acquired using a Recorder64 system (Plexon), and were amplified (x3000), highpass filtered (300Hz), and digitised at 40kHz. Multiunit activity was saved and analysed offline using Offline Sorter (Plexon). After removing artefacts common to all channels, principal component analyses were used to discriminate single units, identified as distinct clusters of spikes within the principal component space, with a clear refractory period in the interspike interval distribution. Spike sorted data were then further analysed using Neuroexplorer (Nex Technologies) and MATLAB R2010a (The Mathworks Inc.), to assess the changes in firing rate of neurons in response to different visual stimuli.

Histology

To establish the location of recording sites, the recording electrode was dipped in fluorescent dye (Cell Tracker CM-DiI; Invitrogen) prior to insertion. In other experiments we have found good correspondence between electrode placements reconstructed using this method and by use of electrolytic lesions [S3]. Following recordings, the mouseõs brain was removed and

post-fixed overnight in 4% paraformaldehyde, prior to cryoprotection for 24 hours in 30% sucrose. 99εm coronal sections were then cut using a sledge microtome, mounted onto glass slides and cover slips were applied using Vectashield (Vector Laboratories, Inc.).

Visual stimuli

Light calibration: Stimuli were measured at the corneal plane using a spectroradiometer (Bentham Instruments Ltd.) between 300-800nm. The effective photon flux for each photopigment was then calculated by weighting spectral irradiance according to pigment spectral efficiency profile as estimated by the pigment spectral efficiency function (derived from a visual pigment template [S4] and ə$_{max}$ values of 365, 480, 498, 508 and 556nm for SWS opsin, melanopsin, rod opsin, MWS opsin and the introduced LWS opsin respectively) mutliplied by an *in vivo* measurement of spectral lens transmission [S5]. The approach is equivalent to that described in Lucas et al (2014) [S6], using spectral efficiency functions available at:
http://lucasgroup.lab.ls.manchester.ac.uk/research/measuringmelanopicilluminance.

Full field visual stimuli: Full field visual stimuli were generated using a custom-made light source (Cairn Research) containing three independently controlled LEDs (ə$_{max}$ at 365nm, 460nm and 600nm). Light from LEDs was combined by a series of dichroic mirrors, passed through a filter-wheel containing neutral-density filters and focused onto opal diffusing glass (5mm diameter; Edmund Optics Inc.) positioned <1mm from the eye. LED intensities and the filter wheel position were controlled with a PC running LabView 8.6 (National Instruments, Ltd.). LEDs were combined to generate two background and stimulus combinations that are summarised in Fig. 1d. The effective photons for ómel-lowô and ódaylightô backgrounds were calculated to be cone isoluminant (14.6 log_{10} LWS-opsin effective photons/cm^2/s (3.0 log_{10} erythropic-lux); 13.9 log_{10} SWS-opsin effective photons/cm^2/s (2.8 log_{10} cyanopic-lux)), but divergent for rods and melanopsin (14.0 and 15.1 log_{10} rod-opsin effective photons/cm^2/s (2.6 and 3.6 log_{10} rhodopic-lux); 14.0 and 15.2 log_{10} melanopsin effective photons/cm^2/s (2.5 and 3.7 log_{10} melanopic-lux)). óMel-lowô and ódaylightô stimuli were generated that were isoluminant to ómel-lowô and ódaylightô backgrounds for rods and melanopsin, but which presented contrast for cone opsins (stimulus = 15.2 log_{10} LWS-opsin effective photons/cm^2/s (3.5 log_{10} erythropic-lux); 14.5 log_{10} SWS-opsin effective photons/cm^2/s (3.3 log_{10} cyanopic-lux)),

Patterned stimuli: Structured images were presented using a custom-made light source containing four independently controlled LEDs (ə$_{max}$ at 405nm, 455nm, 525nm and 630nm; Phlatlight PT-120 Series (Luminus Devices)). LED intensities were controlled with a PC running LabView v.12 (National Instruments, Ltd.). Light from the LEDs was combined by a series of dichroic mirrors (Thorlabs), and directed into a digital mirror device projector (DLP® LightCommanderÊ ; Logic PD Inc.) in place of the original intrinsic light source. Artificial images were generated using Python running PsychoPy Version 1.70.00 [S7].

To expand the area of the retina exposed to the ódaylightô and ómel-lowô spectra additional LED lighting surrounded the projection screen. These LEDs (peak emission at 400nm (Component-Shop), 460nm, 517nm and 630nm (LEDLightsZone)) were arranged in a high density array, and were placed behind Opal Polypropylene (2mm thickness; The Plastic People) to create a diffuse surround. LED intensities were controlled with a Micro-controller

(Arduino UNO, Creative Commons), and matched equivalent photon fluxes of the projection screen.

Although the spectral composition of LEDs in the projector and the surround were thus different from those used for the full field flash, we were able to recreate the effective photon flux for each photopigment of the original ódaylightôand ómel-lowôstimuli based upon spectral irradiance measures at the corneal plane. The radiance of the projected image was also measured, and resulted in intensities of 14.5 and 13.3 $\log_{10}$ melanopsin effective photons/cm^2/sr/s (3.0 and 1.8 $\log_{10}$ melanopic lm/sr), in ódaylightôand ómel-lowôbackgrounds, respectively.

Before presenting structured images with this apparatus, we first confirmed its suitability for exploring melanopsin regulation of vision by duplicating our findings from the full-field flash stimuli. Thus, we projected the cone-isolating flash stimuli under ómel-lowô and ódaylightô conditions across the surface of the screen. The proportion of dLGN units responding to this stimulus was smaller than for full field presentations, as expected given the reduced coverage of the visual scene. In agreement with our earlier data, we found that in *Opn1mw*^{R} mice the amplitude of the mean response was increased in the ómel-lowôcondition (paired t-test of light-evoked change in firing rate, p=0.011). Importantly, *Opn4*$^{-/-}$*;Opn1mw*^{R} animals showed no consistent difference in flash responses between ódaylightô and ómel-lowô conditions (p>0.05 paired t-test).

Subsequently, responses to a range of stimuli were recorded first in the ómel-lowô, then ódaylightô, then the ómel-lowô spectra again, to ensure no temporal drift in response properties. To assay changes in spatial frequency tuning, inverting gratings (Michelson contrast between dark and light bars = 96%) were presented in 4 orientations at two phases (shifted 90°), at 5 different spatial frequencies (0.035-0.56cpd) at 1Hz. To assay changes in temporal frequency tuning, drifting gratings in 8 orientations, at four speeds (0.2-2Hz), were presented at one spatial frequency (0.035cpd). To map receptive fields, bars of positive and negative contrast (Michelson contrast = 72.7%) were presented in the vertical, then horizontal orientation (occupying ~4.5 and 3.6 degrees of the visual field, respectively). These were presented in a pseudo-randomised sequence at 20Hz. Spatial receptive fields were then derived from the responses to this sequence (see below). Responses to a 30s natural movie were also recorded. The natural movie was a recording of mice moving around a behavioural arena, and included movement and looming of different sized objects (subtending visual angles ranging from 0.5 to 36°) at a range of orientations, speeds and contrasts (maximum Michelson contrast = 96 %). The movie lacked differences in colour, and changes in irradiance across time were minimal (standard deviation of irradiance = 5.94%). Responses were undetectable for presentations of de-focussed versions, indicating that most activity was elicited by changes in spatial patterns and object motion.

Environmental light measurements: To gain insight into the range of environmental light levels over which it is appropriate to measure visual responses, we measured the ambient light levels in Manchester, UK (53°21' N, 2°16' W, elevation of 78m), 2 weeks after Summer solstice, for solar angles ranging from ~30 to -9° (equivalent to 1800-2300hrs GMT+1). All solar angles were measured using a spectroradiometer (Bentham Instruments), measuring the relative power in mW/cm^2 at wavelengths between 300-800nm.

Statistical analyses

Receptive field mapping: The spatio-temporal receptive field was derived for each unit by generating the spike triggered average (STA) of responses to bars presented in vertical and horizontal orientations. Black and white bars covering 1/16th of a grey screen were presented at random locations every 50ms (for a duration of 50ms). Bars were preferred to squares on the base of our pilot experiments because they evoked stronger and more repeatable responses, enabling us to obtain more reliable spatiotemporal receptive field estimates and minimize the time of stimulus presentation. The separable spatial and temporal components where then extracted from the raw STA matrix by applying the principal components analysis to rows and columns. The eigenvectors associated with the largest eigenvalues were taken as spatial and temporal kernels. Although the non-separable components of the receptive fields were lost in this procedure, most of energy of the receptive field was preserved with the additional advantage of removing a substantial amount of noise from the raw STA estimates. The spatial receptive field sizes were then separately estimated for the horizontal and vertical dimensions by fitting a Gaussian to the spatial kernels derived from the previous analyses. The receptive field size for individual cells was described as the mean width at half maximum of Gaussians fitted to each dimension. To compare whether spatial receptive fields differed between ódaylightô and ómel-lowô conditions, in each dimension, Gaussians were compared with an F-test, to test whether receptive fields were best fit with a single, or two individual Gaussians.

Classification of direction sensitivity: The direction selectivity index was calculated as described previously [S8]. This was computed as the ratio of $(R_{pref}-R_{null})/(R_{pref}+R_{null})$, where R_{pref} was the response at which the maximum evoked response occurred, and R_{null} was response to movement in the opposite direction to this. Cells exceeding a direction selectivity index of 0.5 were classed as ódirection selectiveô

Natural movie correlation analyses: First, to find cells that showed any kind of reproducible response to the natural movie, we performed a consistency test to the firing rate responses of single units across multiple presentations of the natural movie. Briefly, we computed the average Pearsonõs correlation among pairs of single trial responses. Then we estimated its standard deviation by using a standard bootstrap technique [S9]. We classified a neuronal response as consistent if its average trial-to-trial Pearsonõs correlation was larger than 3 times the standard deviation. Any cell that showed a consistent response, in the ómel-lowô and/or ódaylightô condition, was then included in further analyses. To assess the variation in responses between cells during presentation of the natural movie, we compared the pairwise PSTH Pearsonõs linear correlation (hereafter ósignal correlationö) in the ódaylightô and ómel-lowô conditions. For these cells, we also compared the average trial-by-trial reliability (óautocorrelationö) of responses by computing the Pearsonõs correlation among pairs of single trial responses (time bin = 0.1s) in the ódaylightô and ómel-lowô conditions.

SUPPLEMENTAL REFERENCES

S1. Smallwood, P.M., Olveczky, B.P., Williams, G.L., Jacobs, G.H., Reese, B.E., Meister, M., and Nathans, J. (2003). Genetically engineered mice with an additional class of cone photoreceptors: implications for the evolution of color vision. PNAS *100*, 11706-11711.

S2. Lucas, R.J., Hattar, S., Takao, M., Berson, D.M., Foster, R.G., and Yau, K.W. (2003). Diminished pupillary light reflex at high irradiances in melanopsin-knockout mice. Science *299*, 245-247.

S3. Allen, A.E., Brown, T.M., and Lucas, R.J. (2011). A distinct contribution of short-wavelength-sensitive cones to light-evoked activity in the mouse pretectal olivary nucleus. J Neurosci *31*, 16833-16843.

S4. Govardovskii, V.I., Fyhrquist, N., Reuter, T., Kuzmin, D.G., and Donner, K. (2000). In search of the visual pigment template. Visual Neuroscience *17*, 509-528.

S5. Jacobs, G.H., and Williams, G.A. (2007). Contributions of the mouse UV photopigment to the ERG and to vision. Documenta Ophthalmologica *115*, 137-144.

S6. Lucas, R.J., Peirson, S.N., Berson, D.M., Brown, T.M., Cooper, H.M., Czeisler, C.A., Figueiro, M.G., Gamlin, P.D., Lockley, S.W., O'Hagan, J.B., et al. (2014). Measuring and using light in the melanopsin age. Trends Neurosci *37*, 1-9.

S7. Peirce, J.W. (2007). PsychoPy--Psychophysics software in Python. J Neurosci Methods *162*, 8-13.

S8. Zhao, X., Chen, H., Liu, X., and Cang, J. (2013). Orientation-selective responses in the mouse lateral geniculate nucleus. J Neurosci *33*, 12751-12763.

S9. Efron, B., and Tibshirani, R.J. (1993). An Introduction of the Boostrap, (New York: Chapman and Hall).
